# New Compounds From the Deep‐sea Sponge *Mycale lingua*

**DOI:** 10.1002/cbdv.202503519

**Published:** 2026-02-13

**Authors:** H. Poppy Clark, David Horsley, Amanda Serpell‐Stevens, Tammy Horton, Ann I. Larsson, Emmanuel Tope Oluwabusola, Rainer Ebel, Laurence H. De Clippele, Marcel Jaspars

**Affiliations:** ^1^ Marine Biodiscovery Centre, Department of Chemistry University of Aberdeen Scotland UK; ^2^ Institute of Medical Sciences, Liberty Building, Foresterhill University of Aberdeen Scotland UK; ^3^ National Oceanography Centre Southampton UK; ^4^ Tjärnö Marine Laboratory, Department of Marine Sciences University of Gothenburg Strömstad Sweden; ^5^ School of Biodiversity, One Health & Veterinary Medicine University of Glasgow Scotland UK

**Keywords:** chemical ecology, drug discovery, *Mycale lingua*, natural products

## Abstract

Three compatible solutes and one compound of unknown ecological function were isolated and characterized from the deep‐sea sponge *Mycale lingua* (Bowerbank, 1866), collected from Tisler reef in Norway. These included the first isolation of asterubine and sulcatin from *M. lingua* as well as two new sulcatin analogues, sulcatin B and sulcatin C, which have not previously been reported from natural sources. Compound structures were elucidated through high‐resolution liquid chromatography‐mass spectrometry, and one‐ and two‐dimensional nuclear magnetic resonance spectroscopic methods. All four compounds were tested in tau‐tau aggregation assays to determine if they had potential for the treatment of Alzheimer's disease. No activity was displayed in either the cell‐free or cell‐based tau aggregation assays for any of the compounds.

## Graphical abstract

The focus of this study is the scarcely investigated sponge *Mycale lingua*, found commonly in the North Atlantic Ocean. In total, four compounds are isolated and characterised from three combined M. *lingua* individuals. Structures are fully elucidated through HR‐LCMS and NMR spectroscopy (^1^H, COSY, HSQC, HMBC). Circular dichroism measurements and application of the octant rule are used to assign tentative absolute configurations. None of the compounds display activity against Alzheimer's disease through inhibition of tau‐tau protein aggregation.

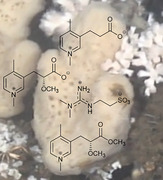

## 1 Introduction

Marine sponges have a global distribution, occur in a wide range of habitats, and some have been found at depths >6000 m [1, 2]. The deep‐sea is an extreme and challenging environment requiring significant adaptation for species survival [3]. The high pressure and salinity associated with these environments require increased presence of, or stronger, osmolytes to regulate the osmolarity of cells [4, 5]. Intracellularly, this role is mainly performed by small zwitterionic organic compounds; unlike common inorganic ions, these do not perturb protein structures/functions, and are hence termed compatible solutes [5]. When maintaining the osmotic pressure of cells, compatible solutes are colligative, but they can provide additional compound‐dependent cytoprotective properties to cells [6]. Therefore, whilst many compatible solutes are shared widely across marine sponges, their quantities and function can vary depending on species and their environment [6, 7, 8, 9].

Interestingly, multiple compatible solutes have demonstrated therapeutic potential against human neurodegenerative diseases, including Parkinson's [10, 11], Huntington's [12], and Alzheimer's disease [13, 14]. In each case, the small organic molecules prevent aggregation of misfolded proteins associated with the diseases, to such an extent that several are in clinical trials [10]. The remarkable chemical diversity displayed by sponges has resulted in the continual discovery of natural products with pharmaceutical potential from investigations into this phylum [15, 16, 17, 18]. However, whilst increased environmental stressors in the deep‐sea can lead to the production of many novel specialized metabolites, the difficulties associated with sampling make deep‐sea sponges an under‐explored source of bioactive compounds [3, 9].

Specialized metabolites of the deep‐sea sponge *Mycale lingua* (Bowerbank, 1866) have previously been investigated in a limited capacity; a proteoglycan displaying immunostimulant activity was patented in 1994 [19], and later, 13 steroidal alkaloids and one lipid were detected in extracts from this sponge [20]. In this study, polar extracts from *M. lingua* collected at Tisler reef in Norway were fractionated and purified, resulting in the isolation of four compounds, including two new compounds and two known compatible solutes.

## 2 Results and Discussion

**FIGURE 1:**
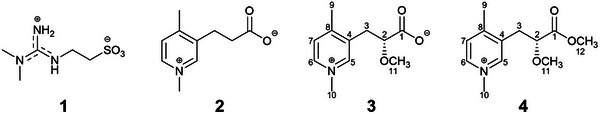
Structures of compounds **1**–**4**, isolated from *Mycale lingua*. The stereochemistri of sulcatin B (**3**) and sulcatin C (**4**) is tentatively assigned based on the octant rule.

The *M. lingua* crude extract (5.66 g) was fractionated using medium‐pressure liquid chromatography. The resulting fractions were purified via reverse‐phase high‐performance liquid chromatography (HPLC) to yield two known compounds, asterubine (**1**) [11, 21, 22, 23], and sulcatin (**2**) [24], and two new compounds, sulcatin B (**3**) and sulcatin C (**4**) (Figure 1). Thus, henceforth sulcatin (**2**) will be referred to as sulcatin A. Asterubine (**1**) and sulcatin A (**2**) were identified through comparison with previously published nuclear magnetic resonance (NMR) and high‐resolution electrospray ionization mass spectrometry (HR‐ESI‐MS) data (Figures S1–10 and Tables S1–2).

**FIGURE 2:**
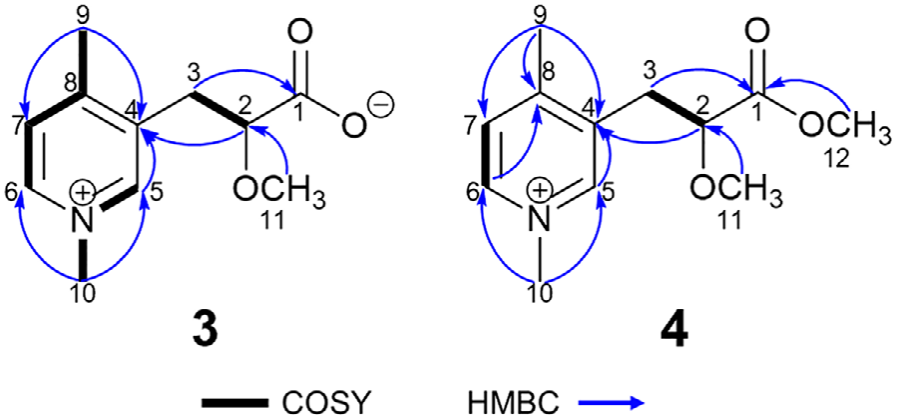
Selected correlated spectroscopy (COSY) and heteronuclear multiple bond coherence (HMBC) data for sulcatin B (**3**) and C (**4**).

The molecular mass of compound **3** was determined based on a protonated molecular ion observed at *m/z* 210.1128 in the high‐resolution ESI‐time of flight‐MS (ESI‐TOF‐MS), corresponding to a molecular formula of C_11_H_16_NO_3_, and requiring five degrees of unsaturation (Figures S11). Analysis of 1D and 2D NMR data (Table 1, Figure 2, and Figures S7–10 and S12–S15) suggested that **3** was a derivative of sulcatin (**2**), except for the presence of an additional methoxy group at C‐2 in **3**. Complete analysis of ^1^H and 2D NMR data showed four methines, one methylene, two methyl, one methoxy, and three quaternary carbon signals (δ_C_ 172.3, C‐1, δ_C_ 136.9, C‐4, and δ_C_ 157.6, C‐8) (Table 1 and Figures S13–S15).

**TABLE 1:** ^1^H‐ and ^13^C nuclear magnetic resonance (NMR) data for sulcatin B (**3**) and C (**4**) in DMSO‐*d*_6_.

Position	3		4	
	^13^C δ^*^	^1^H δ (mult, *J* Hz)	^13^C δ^*^	^1^H δ (mult, *J* Hz)
1	172.3		170.9	
2	77.8	4.06 (dd, 9.4, 4.0)	78.5	4.20 (dd, 9.3, 4.1)
3	32.4	3.19 (dd,14.7, 4.0)	33.1	3.19 (dd, 14.8, 4.1)
3’		3.05 (dd, 14.7, 9.4)		3.07 (dd, 14.8, 9.3)
4	136.9		136.5	
5	144.4	8.77 (br s)	145.2	8.77 (br s)
6	142.5	8.71 (d, 6.3)	143.2	8.72 (d, 6.2)
7	128.0	7.94 (d, 6.3)	128.7	7.94 (d, 6.2)
8	157.6		157.8	
9	19.2	2.57 (s, 3H)	19.9	2.56 (s, 3H)
10	46.9	4.26 (s, 3H)	47.6	4.26 (s, 3H)
11	57.2	3.24 (s, 3H)	58.2	3.24 (s, 3H)
12			52.4	3.72 (s, 3H)

* Obtained from HSQC and HMBC spectra, respectively.

The ^1^H NMR chemical shifts, coupling constants and correlated spectroscopy data (Figure 2 and Figures S7–S9) suggested the presence of a trisubstituted pyridine ring system, with *ortho*‐coupling between H‐6 (δ_H_ 8.71)and H‐7 (δ_H_ 7.94), while the expected *meta‐*coupling between H‐6 and H‐5 (δ_H_ 8.77) was not resolved, but the 1,3,4‐trisubstitution pattern was readily deduced from heteronuclear multiple bond coherence (HMBC) correlations from H‐7 to C‐4 and C‐6 (δ_C_ 142.5), H‐6 to C‐5 (δ_C_ 144.4), C‐7 (δ_C_ 128.0) and C‐8, as well as H‐5 to C‐4, C‐6 and C‐8 (Figure 2). The *para*‐arrangement of the two methyl groups was established by HMBC correlations between H_3_‐9 (δ_H_ 2.57) to C‐4 and C‐7, and H_3_‐10 (δ_H_ 4.26) to C‐5 and C‐6. The chemical shifts for H_3_/C‐10 were deshielded compared to H_3_/C‐9, suggesting that it was adjacent to a quaternary nitrogen of a pyridinium system [24]. The nature and position of the aliphatic side chain was revealed by mutual couplings between H‐2 (δ_H_ 4.06) and H_2_‐3 (δ_H_ 3.19, 3.05), in combination with HMBC correlations between H‐2 and C‐3 (δ_C_ 32.4), C‐4 and C‐11 (δ_C_ 57.2), and from H_2_‐3 to C‐1, C‐4, C‐5, and C‐8 (Figure 2). The positioning of the *O*‐methyl group at C‐2 was corroborated by a diagnostic HMBC correlation between H_3_‐11 (δ_H_ 3.24) and C‐2 (Table S2 and Figure S15).

Compound **4** displayed a protonated molecular ion at *m/z* 224.1283 as determined by high‐resolution ESI‐TOF‐MS, corresponding to a molecular formula of C_12_H_18_NO_3_, and requiring five degrees of unsaturation (Figure S16). The ^1^H, HSQC, and HMBC NMR data (Table 1, Figure 2, and Figures S17–S20) suggested that **4** was the methyl ester of **3**, which was evident from a strong HMBC correlation from H_3_‐12 (δ_H_ 3.72) to C‐1 (δ_C_ 170.9).

**FIGURE 3:**
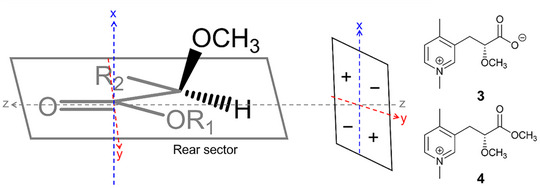
Application of the octant rule to sulcatin B (**3**) (R_1_=−, R_2_=C_8_H_11_N^+^) and sulcatin C (**4**) (R_1_=CH_3_, R_2_=C_8_H_11_N^+^), and their resulting predicted stereochemistry.

Compounds **3** and **4** both possess chiral centers at C‐2. The absolute stereochemistry of some carbonyl‐containing compounds can be determined through their measured Cotton effect and the octant rule [25, 26, 27]. The octant rule is an empirical set of guidelines for chiral saturated alkyl ketones, derived from the symmetry of the orbitals in the n −> π* transition observed during CD spectroscopy. The space surrounding the carbonyl chromophore is divided into eight regions (octants) by three planes (zx, zy, and xy) (Figure 3). The location of substituents within these octants determines their contribution to the Cotton effect of the molecule. Substituents within the octant planes do not contribute to the Cotton effect [25, 27]. The results from circular dichroism analysis (Figure S21) reveal that both compounds **3** and **4** display positive Cotton effects. Implementing the octant rule, therefore, suggests that these compounds possess S stereochemistry (Figure 3). On the basis of the evidence provided, the structures of compounds **3** and **4** were established as depicted, with tentative assignment of their stereochemistry, and identified as new natural products for which the trivial names sulcatin B (**3**) and C (**4**), respectively, are suggested.

As asterubine (**1**) and sulcatin A (**2**) have been characterized previously, some of their bioactivity is known. Asterubine (**1**) has previously been isolated from Australian sponges [11, 23] and a Japanese sea‐star [21]. It is non‐cytotoxic, promotes terrestrial plant growth, and has displayed binding activity to the Alzheimer's and Parkinson's disease‐associated protein α‐synuclein [11, 21, 23]. Sulcatin A (**2**) has previously been isolated from a Mediterranean tunicate and showed antiproliferative activity against monocyte/macrophage cells [24]. The zwitterionic nature of asterubine (**1**) and sulcatins A and B (**2** and **3**) suggests that they act as compatible solutes within *M. lingua*, helping cells of the holobiont to regulate their osmolarity. The change from a carboxylic acid to an ester moiety from sulcatin B (**3**) to sulcatin C (**4**) makes sulcatin C (**4**) permanently positively charged, and therefore less likely to behave as a compatible solute. It should be noted that the methoxy groups present in sulcatin B (**3**) and sulcatin C (**4**) could have resulted from hydroxy groups reacting with methanol during the extraction process. Insufficient material prevented the repetition of this extraction with ethanol.

The expected functional roles of sulcatin A (**2**) and sulcatin B (**3**) suggest that they may display activity against protein aggregation in neurodegenerative diseases, as has been seen for other compatible solutes [10]. Therefore, asterubine (**1**), sulcatin A (**2**), sulcatin B (**3**), and sulcatin C (**4**) were tested for activity against the aggregation of tau protein, associated with Alzheimer's disease and other tauopathies, using cell‐free and cell‐based assays [28]. All compounds were inactive in both assays at the maximum concentration tested (Figures S22–S25).

## 3 Conclusion

In total, four compounds were isolated from the deep‐sea sponge *M. lingua*. These included two known compounds, asterubine and sulcatin A, as well as two new compounds, sulcatins B and C. Asterubine, sulcatin A, and sulcatin B are predicted to be compatible solutes within the sponge. Sulcatin C is predicted to perform a separate functional role from the compatible solutes, which may be elucidated through further bioactivity testing of the compound. Whilst none of the compounds were active against tau‐tau cell‐free or cell‐based aggregation, this does not exclude them from other potential modes of action to combat neurodegenerative diseases.

## 4 Experimental

### 4.1 General Experimental Procedures

UV spectra were recorded on a Shimadzu 2600i 2 (UV‐2600 Series, Japan) using a 1 cm plastic cell. HR‐ESI‐MS data were obtained using a Bruker MAXIS II Q‐ToF mass spectrometer (Coventry, UK), coupled to an Agilent 1290 UHPLC. The UHPLC used a Phenomenex Kinetex XB‐C18 (2.6 mM, 100 × 2.1 mm) column and UV detection between 200‐400 nm; a flow rate of 1 mL/min was used with a mobile phase gradient of LC‐MS grade H_2_O (0.1% formic acid): acetonitrile (0.1% formic acid) from 5%–95% in 10 min and isocratic elution at 95% for 2 min. Q‐ToF MS parameters were: mass range m/z 100–2000, capillary voltage 4.5 kV, nebulizer gas 4.0 bar, dry gas 9.0 L/min, and temperature of 150°C. MS/MS experiments were conducted under auto MS/MS scan mode with no step collision; MS acquisition occurred for 12 min of the run. Molecular formulae were generated using the Bruker Data Analysis software, and their associated errors calculated [29].

1D and 2D NMR spectra were recorded on a Bruker AVANCE III spectrometer (Coventry, UK) at 400 and 600 MHz; the latter has a liquid nitrogen‐cooled Prodigy cryoprobe. All ^1^H and ^13^C chemical shifts were reported in the standard δ notation of ppm and referenced to the solvent peaks.

Fractionation was carried out on a Flash Pure EcoFlex BUCHI C18 50 µm spherical 80 g column. Purification was completed using an Agilent 1260 II Infinity semi‐preparative HPLC system (Germany) equipped with a quaternary pump, photodiode array detector (DAD), Waters Sunfire reversed‐phase column C_18_ (5 µm, 10 × 250 mm), and a mobile phase solvent gradient between 95:5 % (H_2_O/MeOH).

Optical rotation was measured using an ADP 410 automatic digital Polarimeter containing LED optics and a photodiode detector (Bellingham + Stanley, UK) and a 5 cm cell at 20°C.

The Cotton effect of molecules was measured using a Circular Dichroism Spectrometer MOS‐500 (France), with a 1 cm plastic cell and 1 mL of CH_3_OH as solvent.

### 4.2 Sample Collection and Identification

The three sponge samples were collected from between 112 and 134 m at Tisler reef in Norway (58.99967 N, 10.97067 E) in October 2020, using an OceanModules remotely operated vehicle (ROV V8 Sii, P/N: 02/00100‐01, S/N: 011), deployed from the RV Nereus. The samples were freeze‐dried using a Ninolab Heto LyoPro 6000 freeze‐dryer (Sweden) and stored at 4°C. They were identified as *M. lingua* by Dr Tammy Horton and Amanda Serpell‐Stevens of the National Oceanographic Centre (NOC), Southampton, UK. Voucher specimens for the three samples (DISCOLL‐TR‐MTA‐POR‐001, DISCOLL‐TR‐MTB‐POR‐002, and DISCOLL‐TR‐MTH‐POR‐008) are held at the NOC as part of The Discovery Collections (DISCOLL, NOC, UK; http://grscicoll.org/institution/national‐oceanography‐centre‐southampton; https://www.noc.ac.uk/facilities/discovery‐collections).

### 4.3 Extraction and Isolation

The sponge samples were combined, macerated, and extracted with MeOH (3 × 1L) followed by CH_2_Cl_2_ (1 × 1 L). The combined organic extracts (5.66 g) were dried in vacuo before being re‐dissolved in 30 mL of H_2_O and separated via medium‐pressure liquid chromatography with a Flash Pure EcoFlex BUCHI C18 50 µm spherical 80 g column and 10 mL/min flow rate (H_2_O/MeOH solvent system). The solvent gradient was from 20%–100% MeOH in 20% steps for 20 min each. The 8 fractions (FR1‐8) were dried and weighed. HR‐LCMS analysis showed that FR2‐4 (0.70 g, 0.14 g, 0.04 g, respectively) contained the same compounds. The large salt component of FR2 was removed by dissolving the sample in cold MeOH; the organic solution was transferred and dried (0.26 g). Each fraction was then further separated on a Sunfire reversed‐phase column using a H_2_O/MeOH staged solvent system with 95:5% for 16 min, and 85:25% for 15 min, followed by a gradient from 85:25% to 0:100% in 5 min. This resulted in up to 12 fractions (FH1‐12) per MPLC FR fraction. The FR‐FH fractions were purified using reversed‐phase HPLC and a H_2_O/MeOH (with TFA) staged solvent system with 95:5% for 30 min, and 75:25% for 10 min followed by a gradient from 75:25% to 0:100% in 5 min to yield compounds **1** (2.5 mg), **2** (1.2 mg), **3** (1.3 mg), and **4** (1.5 mg).

Sulcatin B (**3**) white solid. Yield = 1.3 mg. UV (MeOH), λ_max_ 262 nm. ^1^H, ^13^C, and 2D NMR data (DMSO‐*d*_6_) are given in Table 1, Table S2, and Figures S12–15. HR‐ESI‐MS: *m/z* 210.1128 [M]^+^ (Figure S11), calc. for C_11_H_16_NO_3_, Δ = 1.6 ppm. The specific rotation was ${\left[\alpha \right]}_{D}^{20}$ −0.13 (c 7.5 × 10^−4^, CH_3_OH).

Sulcatin C (**4**) white solid. Yield = 1.5 mg. UV (MeOH), λ_max_ 262 nm.^1^H, ^13^C, and 2D NMR data (DMSO‐*d*_6_) are given in Table 1, Table S2, and Figures S17–20. HR‐ESI‐MS: *m/z* 224.1283 [M]^+^ (Figure S16), calc. for C_12_H_18_NO_3_, Δ = 0.8 ppm. The specific rotation was ${\left[\alpha \right]}_{D}^{20}$ −0.08 (c 5.0 × 10^−4^, CH_3_OH).

### 4.4 Tau Aggregation Assays

Compounds **1**–**4** were tested in cell‐free (B_50_) and cell‐based (EC_50_) tau‐tau aggregation inhibitor assays as previously described [28]. Compounds were made up at 100 mM stocks in dimethyl sulfoxide (DMSO). A 10 mM working stock was made in DMSO, and both were stored at −20°C until testing. Each assay was run twice for each compound. In the cell‐free aggregation assay, compounds were tested for activity up to a concentration of 500 µM, using 1,9‐dimethylmethylene blue (DMMTC) as a positive control. In the cell‐based aggregation assay, compounds were tested on two separate clones (21C1 and 25A9) at 2 µM and 20 µM. Each plate included an untreated control, and MTC was used as a positive control compound.

## Author Contributions

**Ann I. Larsson**: organized sampling logistics and permits, **Laurence H. De Clippele**: collected the samples, and **Amanda Serpell‐Stevens and Tammy Horton**: identified them. **H. Poppy Clark**: performed the extraction, isolation, and purification of the compounds. **Emmanuel Tope Oluwabusola**: assisted in the purification process. **H. Poppy Clark, Emmanuel Tope Oluwabusola, and Rainer Ebel**: elucidated the compound structures. **H. Poppy Clark**: elucidated the absolute configuration of compounds. **David Horsley**: performed the biological assays. **Marcel Jaspars**: designed the experiment and provided supervision. **H. Poppy Clark**: drafted the initial manuscript, which was edited and approved by all authors.

## Conflicts of Interest

The authors declare no conflicts of interest.

## Funding

H. Poppy Clark is supported by the Biotechnology and Biological Sciences Research Council EASTBIO Doctoral Training Programme (BB/M010996/1). Tammy Horton and Amanda Serpell‐Stevens received funding from a DEFRA GCBC Grant ‘DEEPEND’. The research leading to the results in this manuscript received funding from the ASSEMBLE Plus AmpLOPHELIA project (Grant Agreement No. 730984).

## Supporting information




**Supporting File 1**: cbdv70977‐sup‐0001‐SuppMat.docx

## Data Availability

The authors have nothing to report.
