# Supplementary material for: New Compounds From the Deep‐sea Sponge Mycale lingua
Source: Chem Biodivers. 2026 Feb 13;23(2):e03519. doi: 10.1002/cbdv.202503519 (PMC12903071; doi:10.1002/cbdv.202503519)
Supplement: Supplementary file 1 — Supporting File 1: cbdv70977‐sup‐0001‐SuppMat.docx [file CBDV-23-e03519-s001.docx]

**SUPPLEMENTARY MATERIAL: New compounds from the deep-sea sponge *Mycale lingua***

H. Poppy Clark,^1*^ David Horsley,^2^ Amanda Serpell-Stevens,^3^ Tammy Horton,^3^ Ann I. Larsson,^4^ Emmanuel Tope Oluwabusola,^1^ Rainer Ebel,^1^ Laurence H. De Clippele,^5^ and Marcel Jaspars.^1*^

1. Marine Biodiscovery Centre, Department of Chemistry, University of Aberdeen, Old Aberdeen, AB24 3UE, Scotland, United Kingdom

2. Institute of Medical Sciences, Liberty Building, Foresterhill, University of Aberdeen, AB25 2ZP, Scotland, United Kingdom

3. National Oceanography Centre, European Way, Southampton, SO14 3ZH, United Kingdom

4. Tjärnö Marine Laboratory, Department of Marine Sciences, University of Gothenburg, Strömstad, Sweden

5. School of Biodiversity, One Health & Veterinary Medicine, University of Glasgow, Bearsden Road, G61 1QH, Scotland, United Kingdom

*Corresponding authors: H. Poppy Clark ([h.clark.21@abdn.ac.uk](mailto:h.clark.21@abdn.ac.uk)) and Marcel Jaspars ([m.jaspars@abdn.ac.uk](mailto:m.jaspars@abdn.ac.uk))

**Table of contents**

**Figure S1, Table S1, Figure S2** HRESIMS, full NMR data and ^1^H-NMR spectra of **1** 3

**Figure S3-4** HSQC and COSY NMR spectra of **1** 4

**Figure S5-6** HMBC NMR data of **1** and HRESIMS of **2** 5

**Figure S7-8** ^1^H and HSQC NMR spectra of **2** 6

**Figure S9-10** COSY and HMBC NMR spectra of **2** 7

**Figure S11-12** HRESIMS and ^1^H-NMR spectra of **3** 8

**Figure S13-14** HSQC and COSY NMR spectra of **3** 9

**Figure S15-16** HMBC NMR spectra of **3** and HRESIMS of **4** 10

**Figure S17-18** ^1^H and HSQC NMR spectra of **4** 11

**Figure S19-20** COSY and HMBC NMR spectra of **4** 12

**Table S2** Full NMR data for **2-4** 13

**Figure S21-22** CD spectra of **3-4** and first cell-free tau-tau binding results for **1**-**4** 14

**Figure S23-24** Second cell-free tau-tau binding results for **1**-**4** and western blot

results for cell-based tau-tau assay for **1**-**4** 15

**Figure S25** Tau band ratios from cell-based tau-tau binding assay for **1**-**4** 16


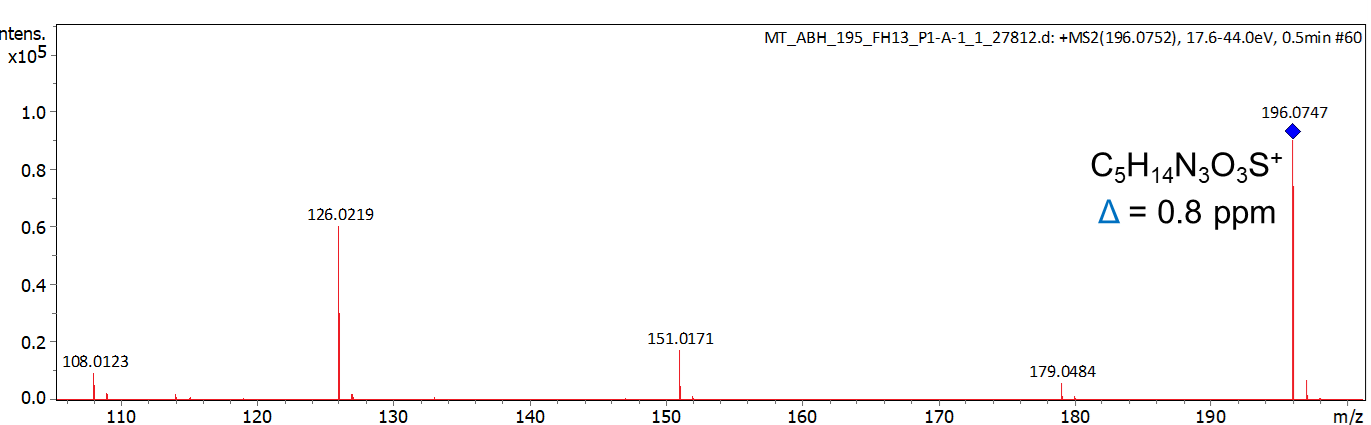


**Figure S1:** QTOF-(+)-HRMS/MS of **1** (*m/z* 196.0752, [M+H]^+^, calc. for C_5_H_14_N_3_O_3_S, Δ=0.8 ppm).

**Table S1:** 400 MHz ^1^H- and ^13^C-NMR data for asterubine (**1**) in DMSO-*d*_6_.

| **Number** | **^13^C δ^*^** | **^1^H δ (mult, *J* Hz)** | **COSY** | **HMBC**  **(^1^H →^13^C)** |
| --- | --- | --- | --- | --- |
| 1 | 49.8 | 2.72 (t, 6.2, 2H) | 2 | 2 |
| 2 | 39.6 | 3.43 (q, 11.8, 5.7, 2H) | 1 | 1,3 |
| 3 | 155.8 |  |  |  |
| 4 | 38.3 | 2.93 (s, 6H) | 2 | 3 |

* obtained from HSQC and HMBC spectra, respectively

**
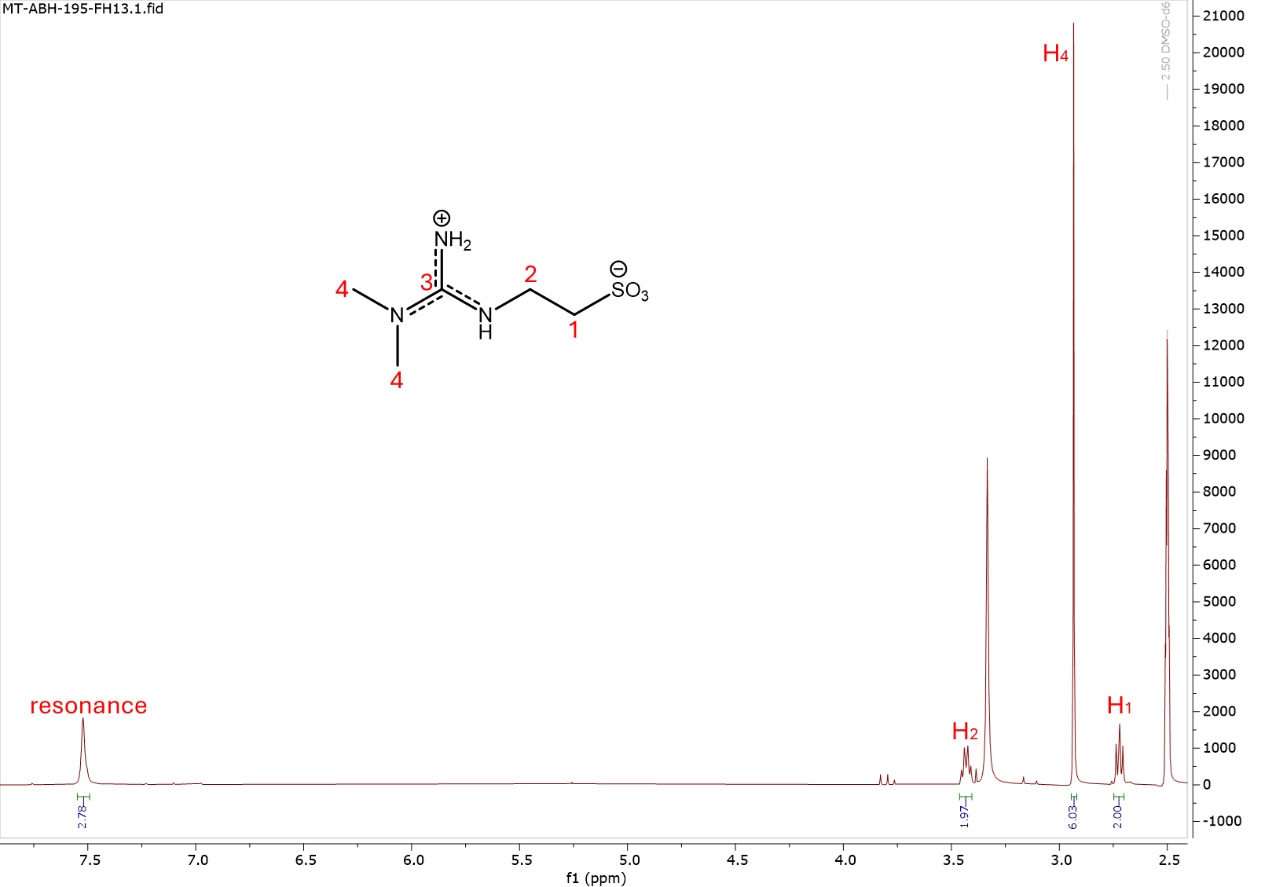
Figure S2:** ^1^H-NMR spectrum of **1** in DMSO-*d*_6_ at 400 MHz.


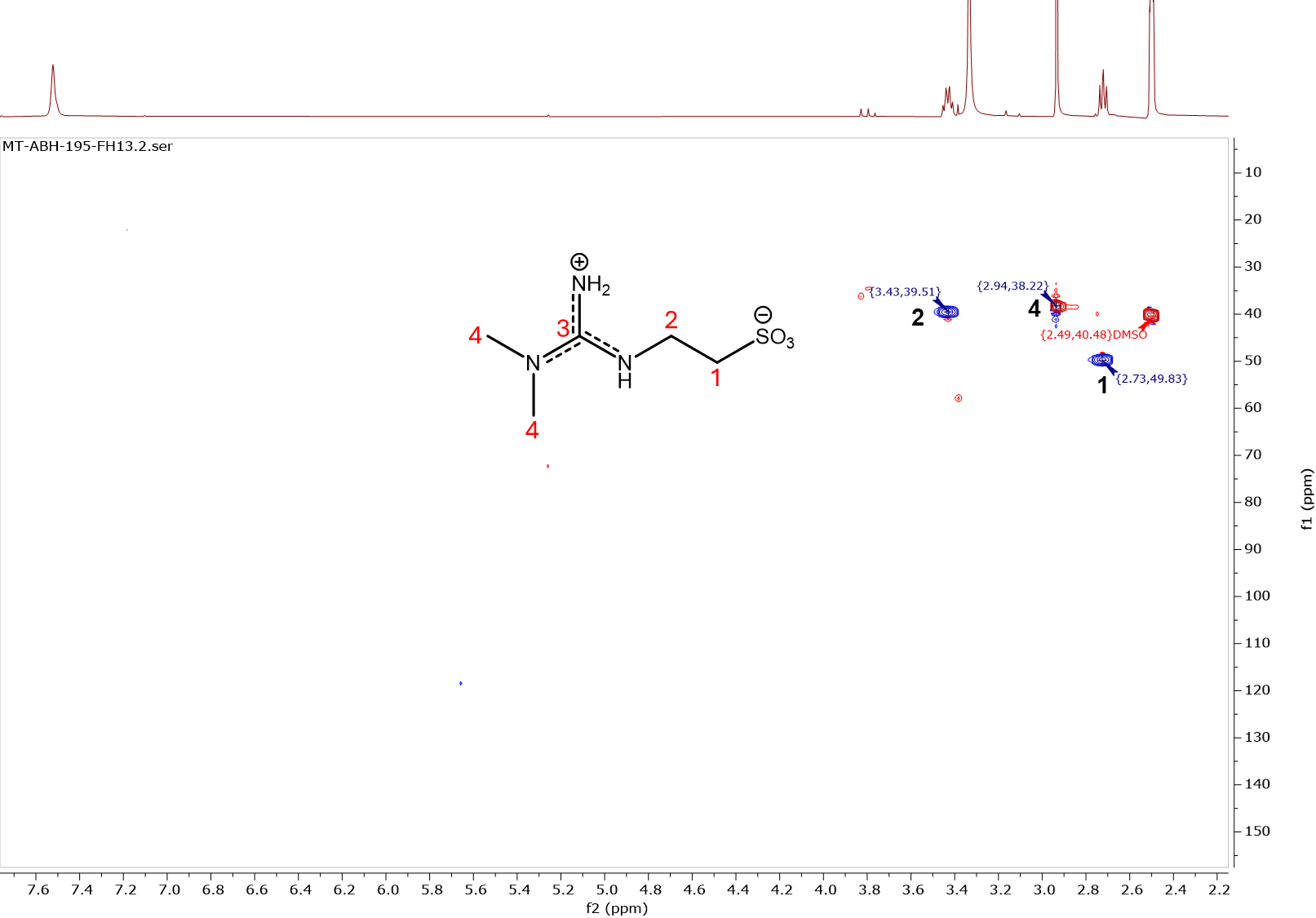


**Figure S3:** HSQC NMR spectrum of **1** in DMSO-*d*_6_ at 400 MHz.


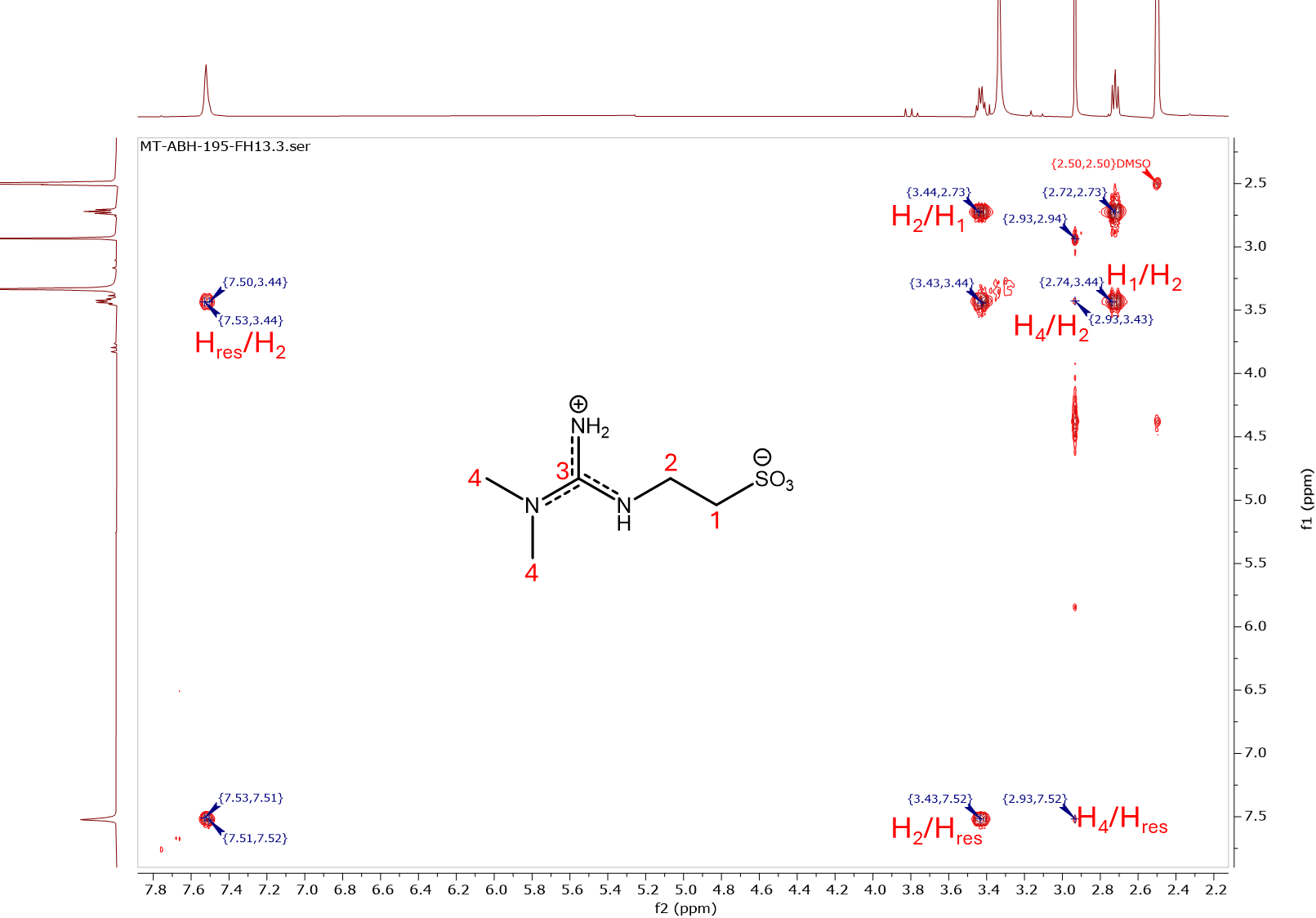


**Figure S4:** COSY NMR spectrum of **1** in DMSO-*d*_6_ at 400 MHz.


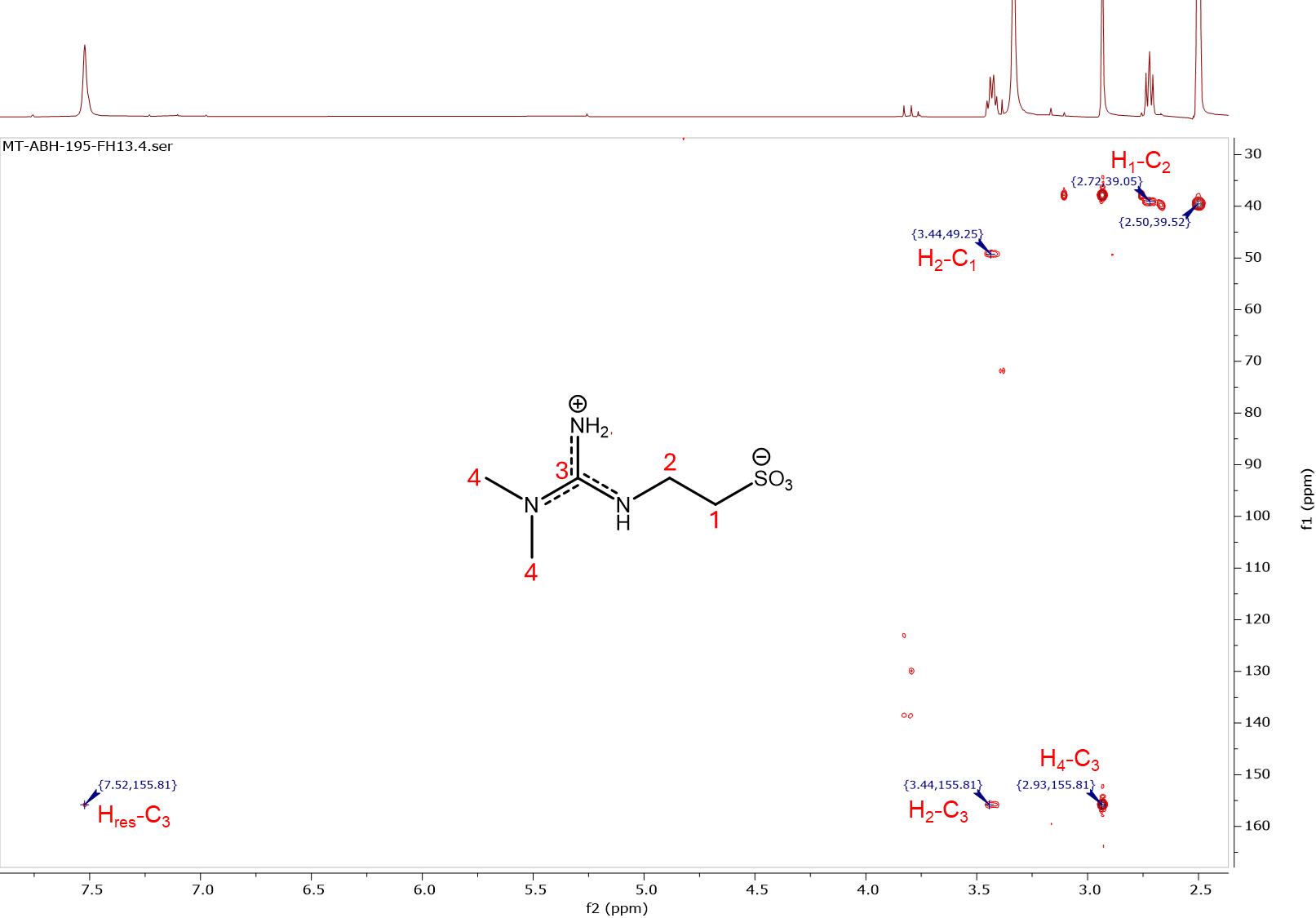


**Figure S5:** HMBC NMR spectrum of **1** in DMSO-*d*_6_ at 400 MHz.


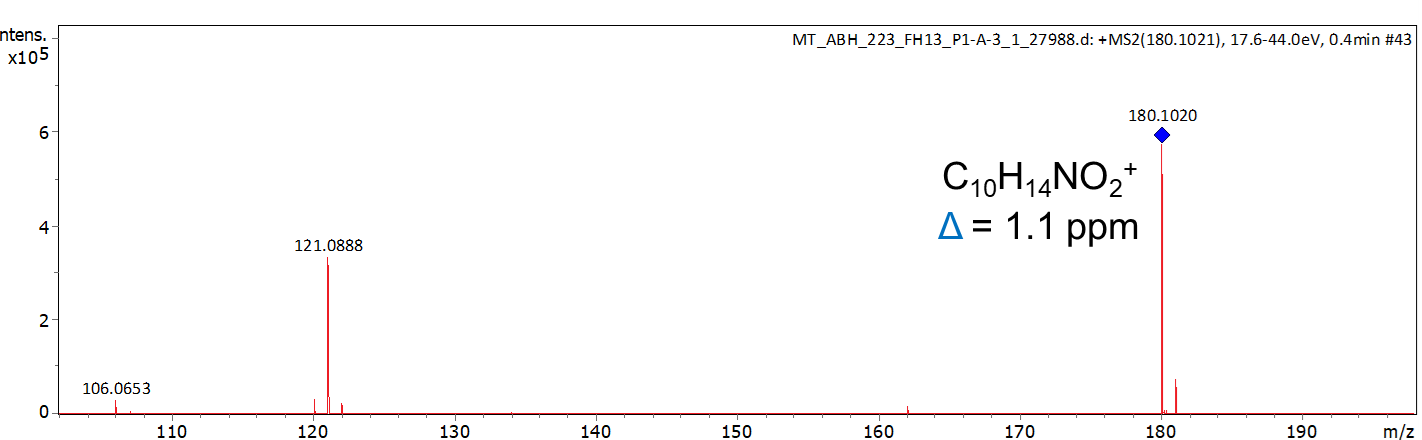


**Figure S6:** QTOF-(+)-HRMS/MS of **2** (*m/z* 180.1021, [M+H]^+^, calc. for C_10_H_14_NO_2_, Δ=1.1 ppm).


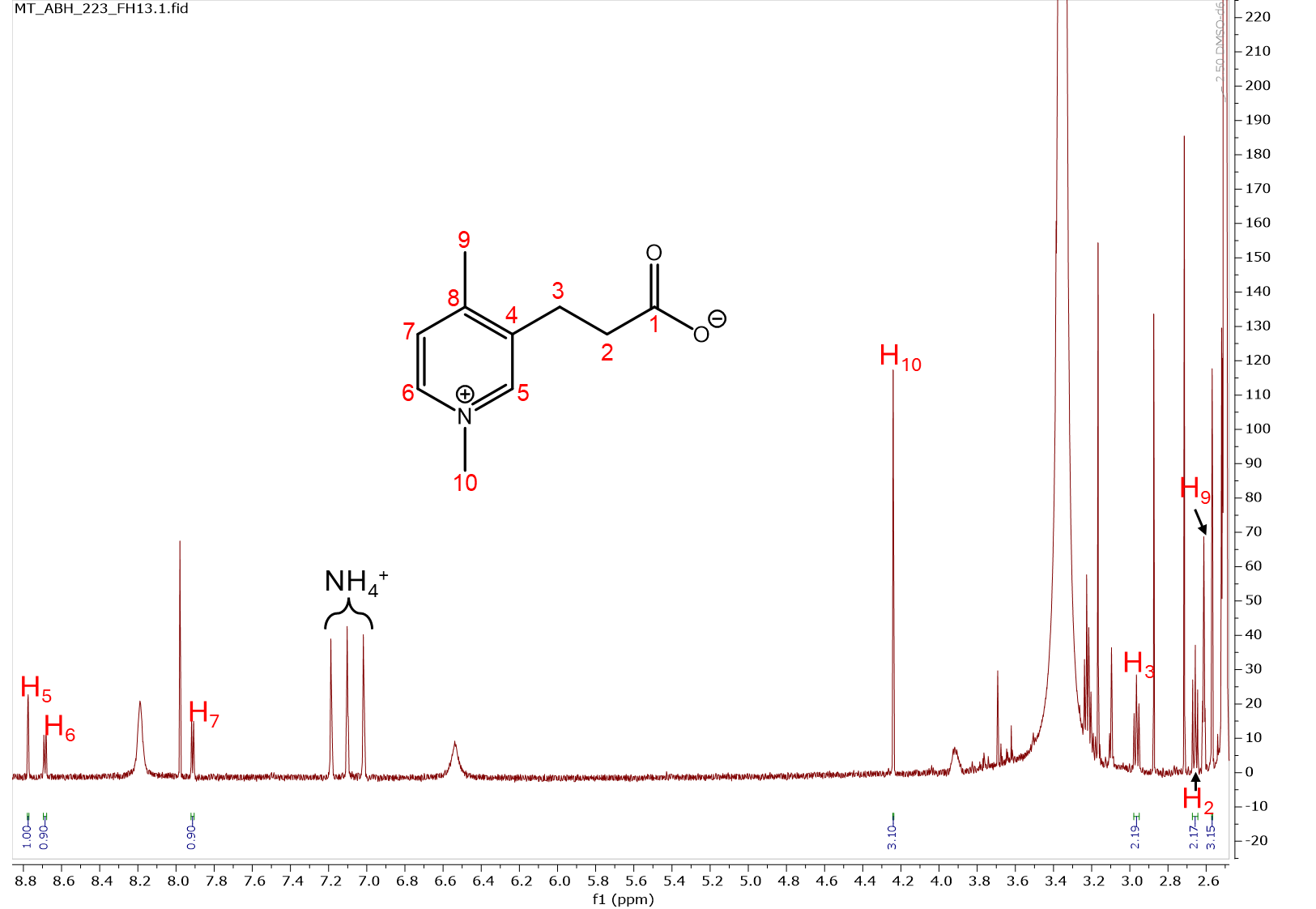


**Figure S7:** ^1^H-NMR spectrum of **2** in DMSO-*d*_6_ at 600 MHz.


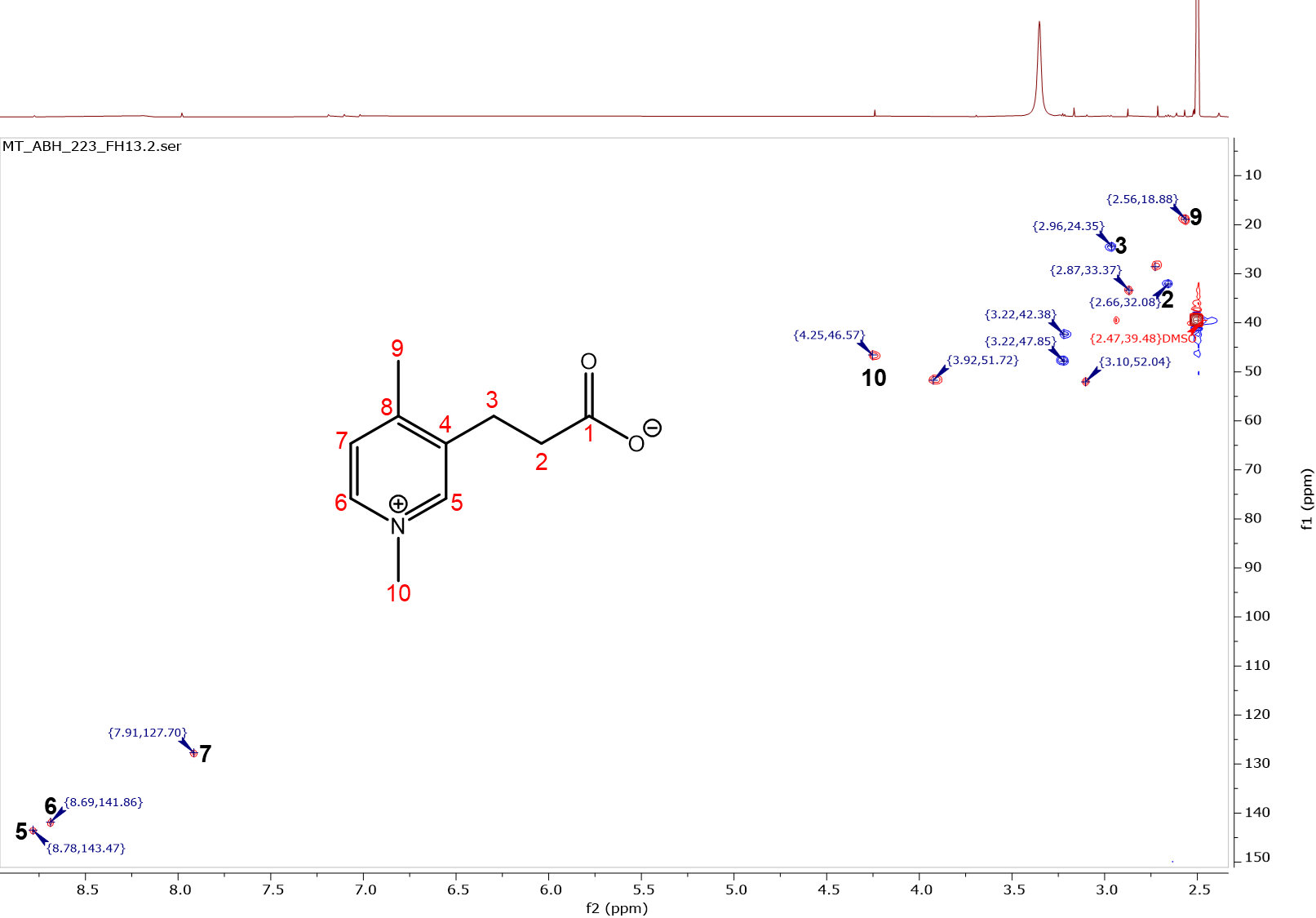


**Figure S8:** HSQC NMR spectrum of **2** in DMSO-*d*_6_ at 600 MHz.


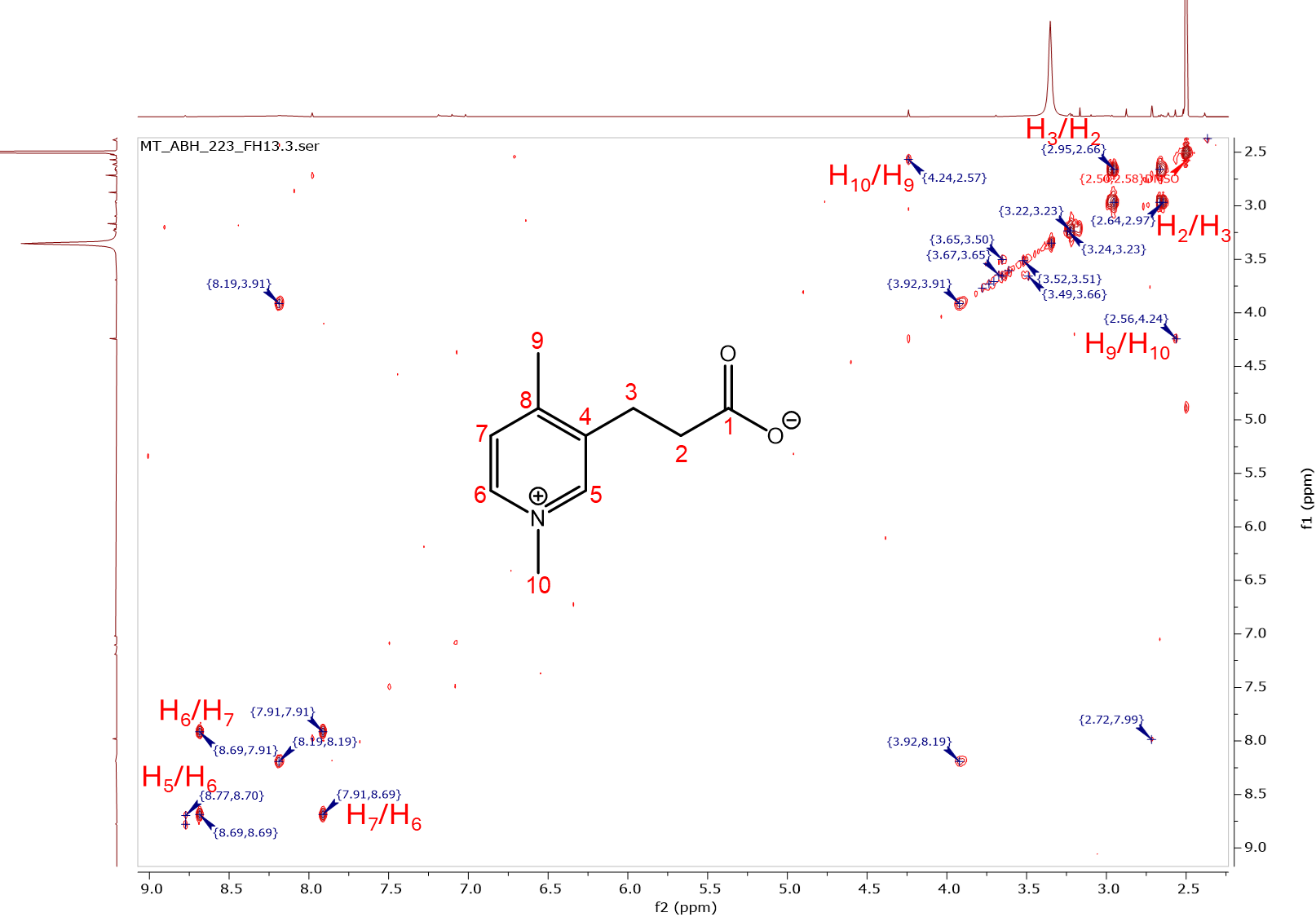


**Figure S9:** COSY NMR spectrum of **2** in DMSO-*d*_6_ at 600 MHz.


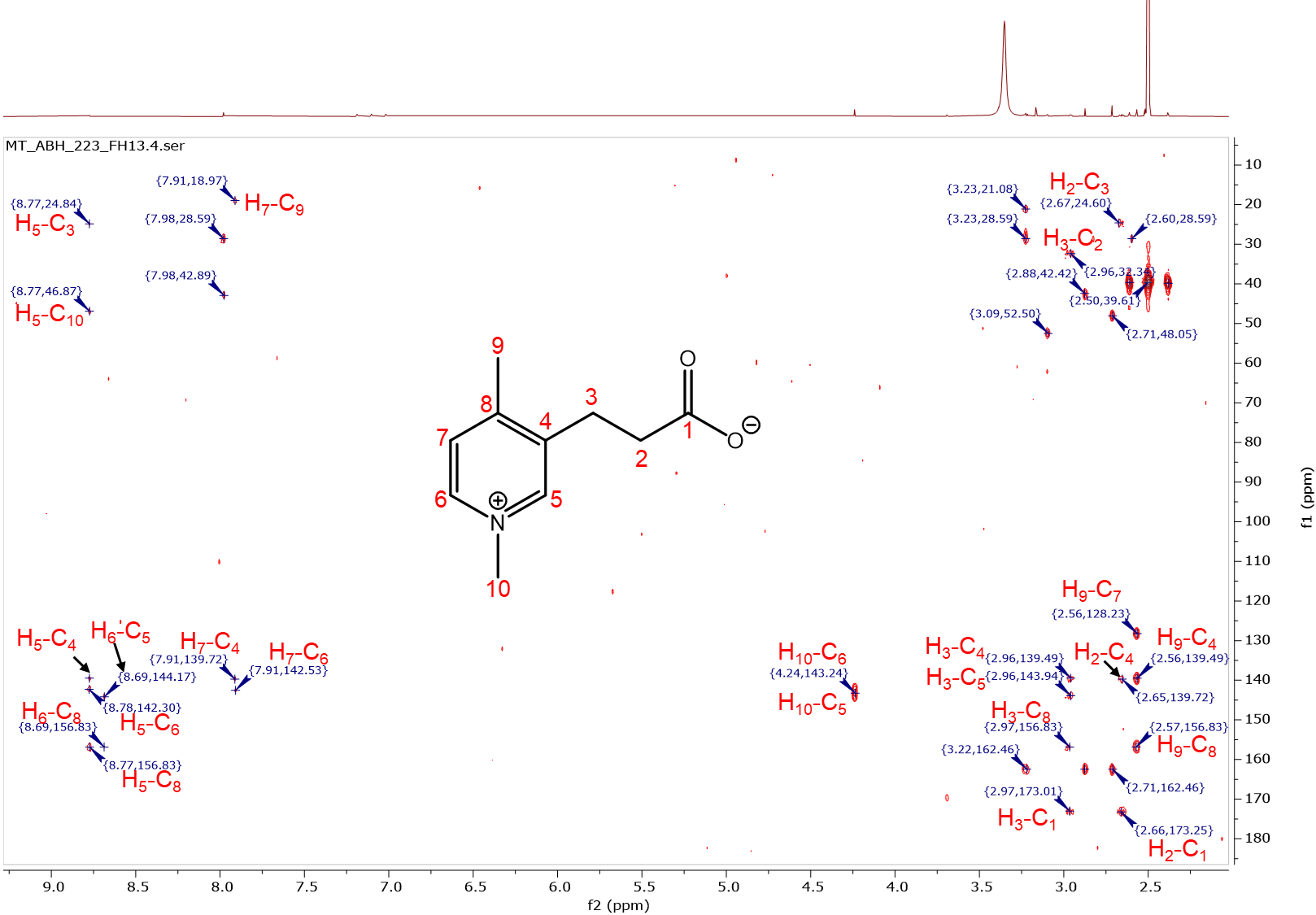


**Figure S10:** HMBC NMR spectrum of **2** in DMSO-*d*_6_ at 600 MHz.


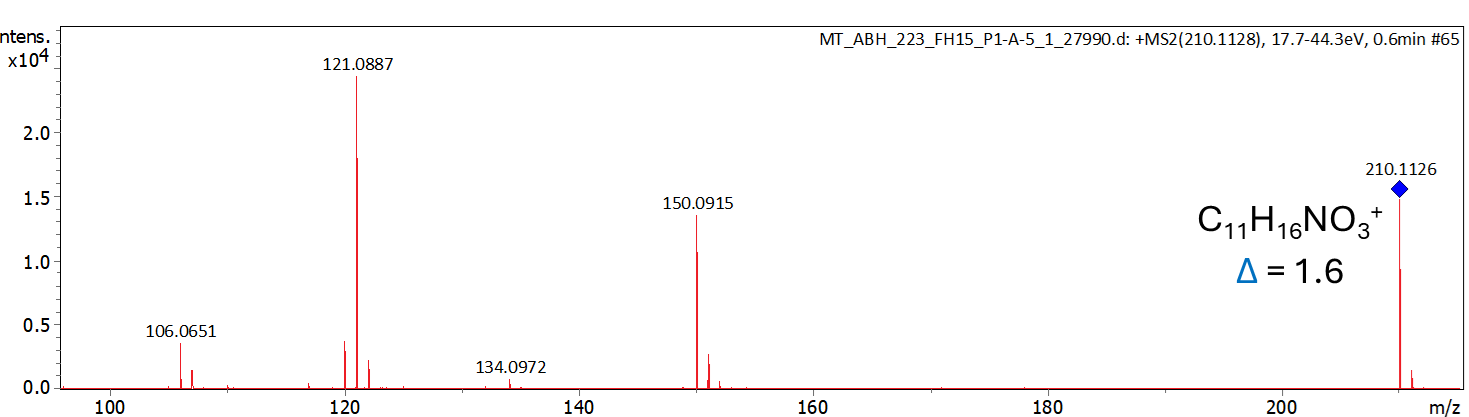


**Figure S11:** QTOF-(+)-HRMS/MS of **3** (*m/z* 210.1128, [M+H]^+^, calc. for C_11_H_16_NO_3_, Δ = 1.6 ppm).


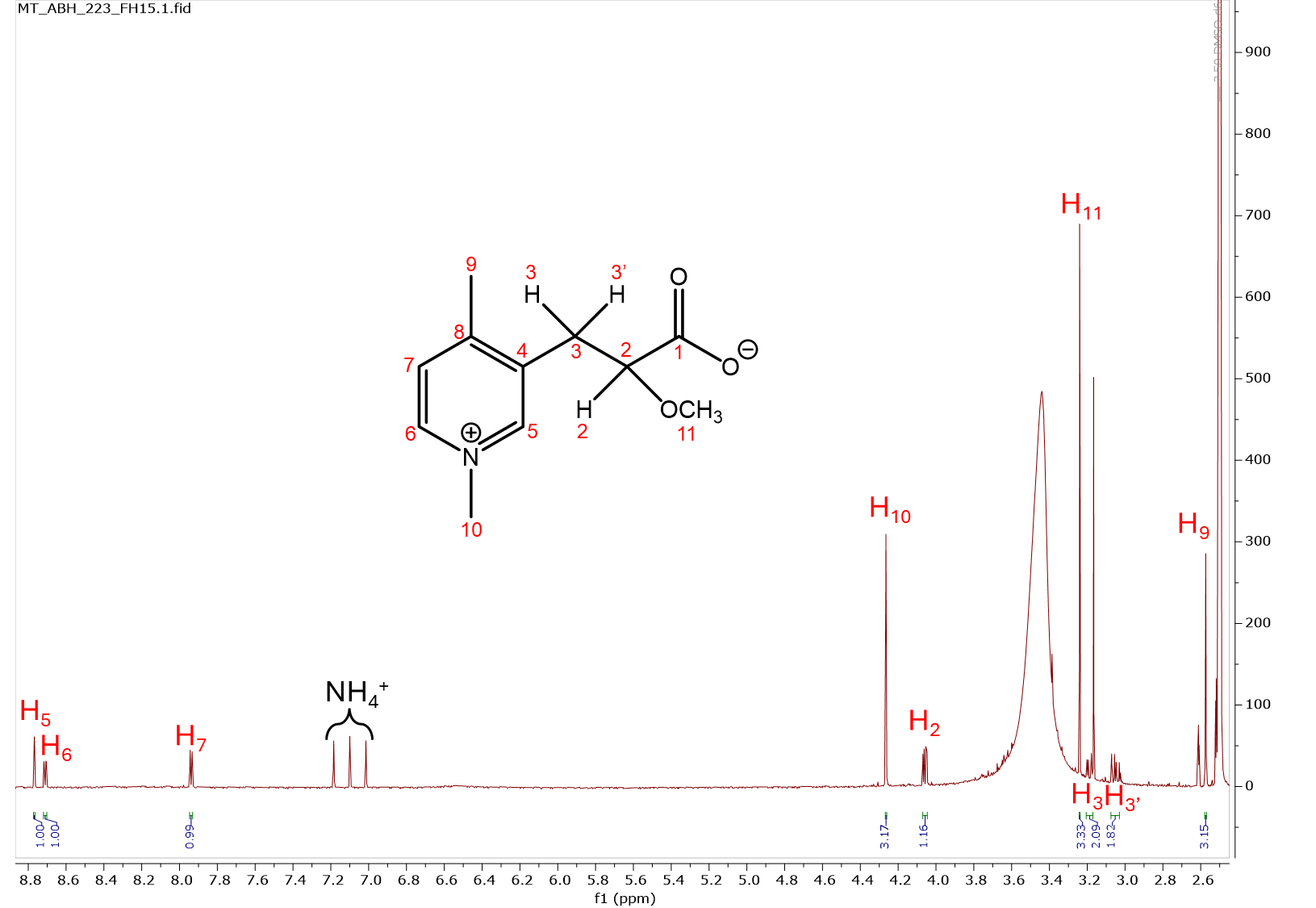


**Figure S12:** ^1^H-NMR spectrum of **3** in DMSO-*d*_6_ at 600 MHz.


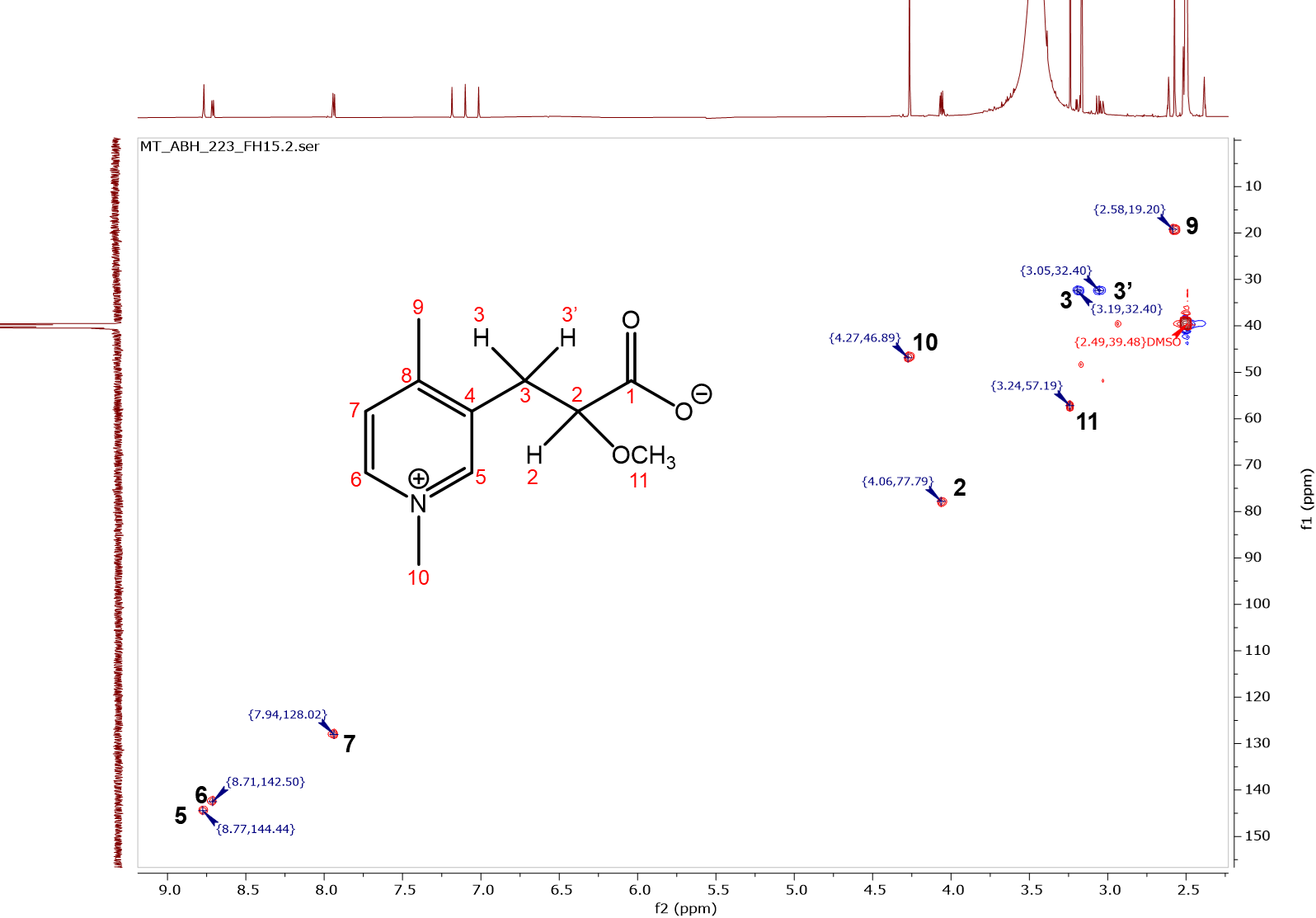


**Figure S13:** HSQC NMR spectrum of **3** in DMSO-*d*_6_ at 600 MHz.


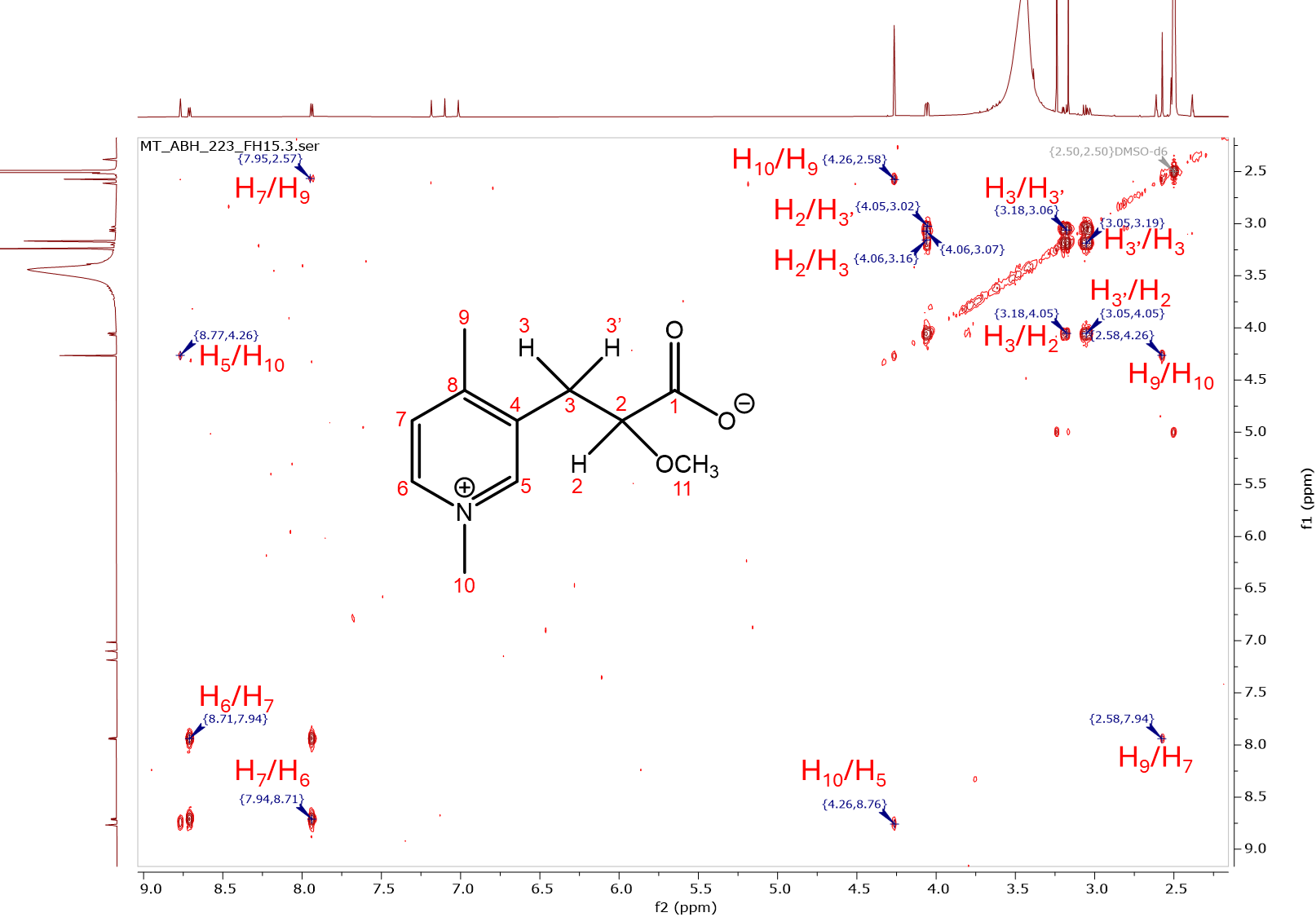


**Figure S14:** COSY NMR spectrum of **3** in DMSO-*d*_6_ at 600 MHz.


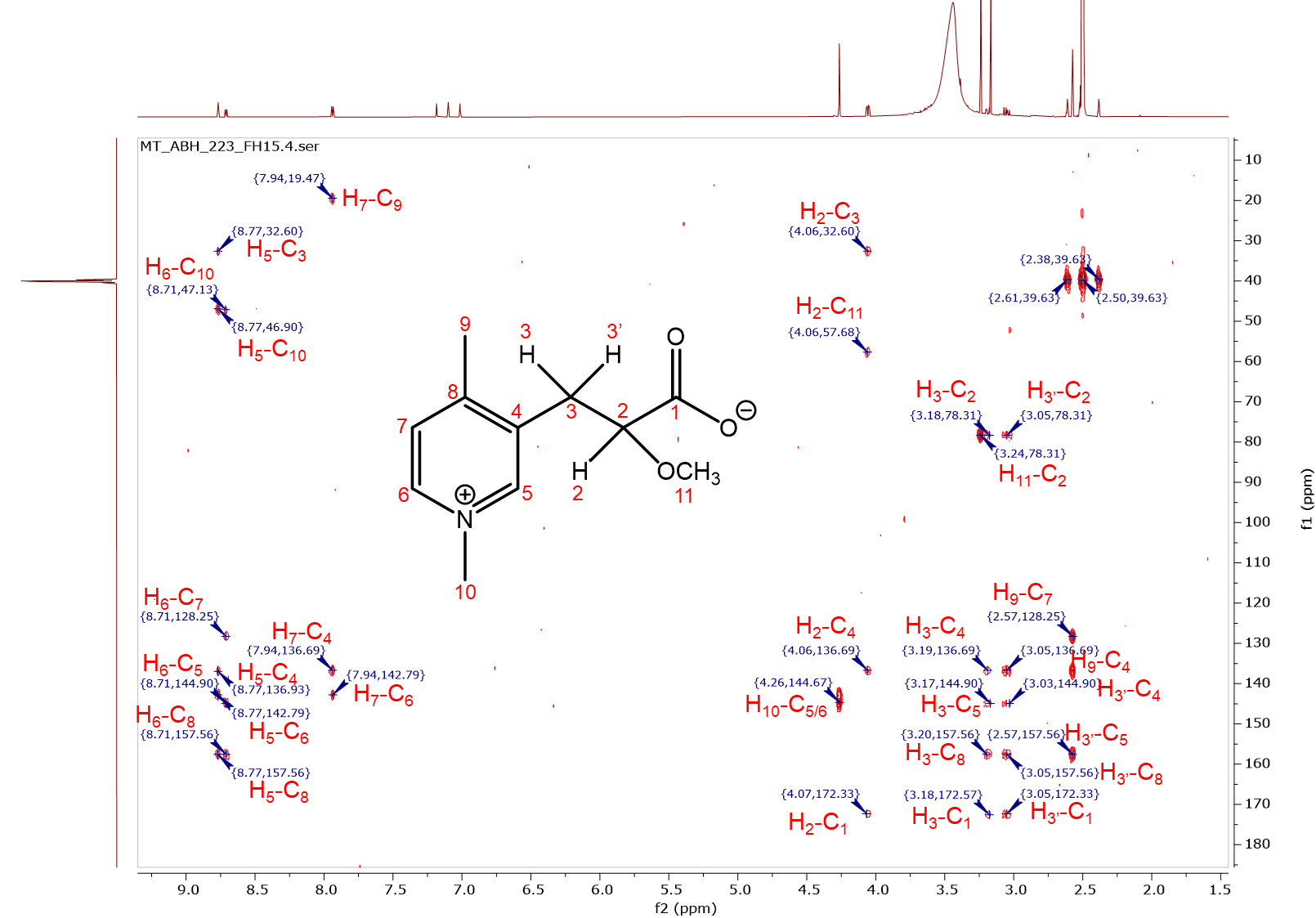


**Figure S15:** HMBC NMR spectrum of **3** in DMSO-*d*_6_ at 600 MHz.


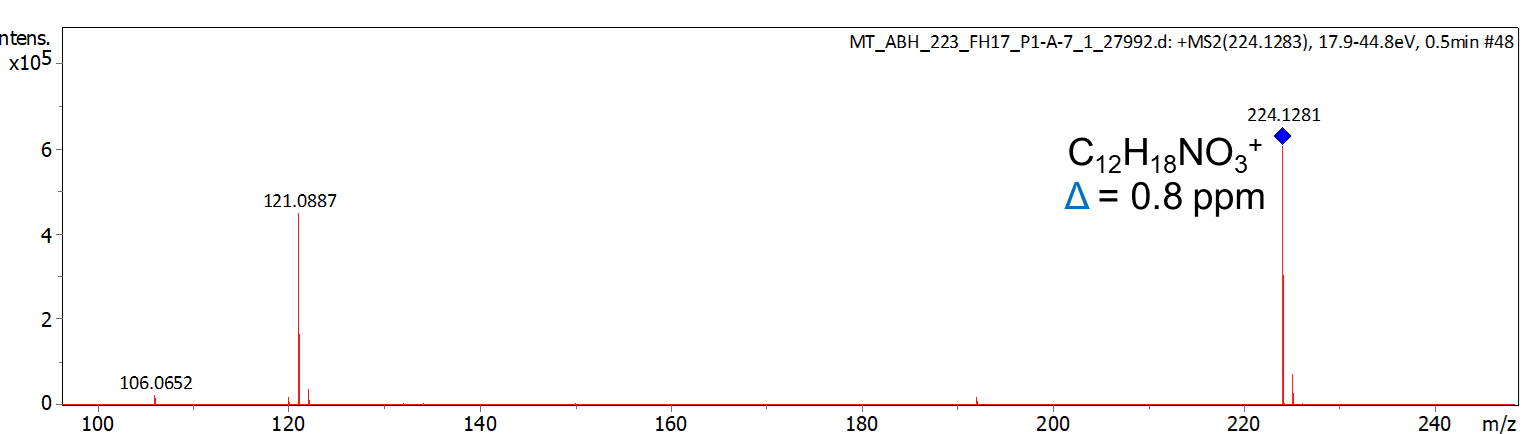


**Figure S16:** QTOF-(+)-HRMS/MS of **4** (*m/z* 224.1283, [M]^+^, calc. for C_12_H_18_NO_3_, Δ = 0.8 ppm).


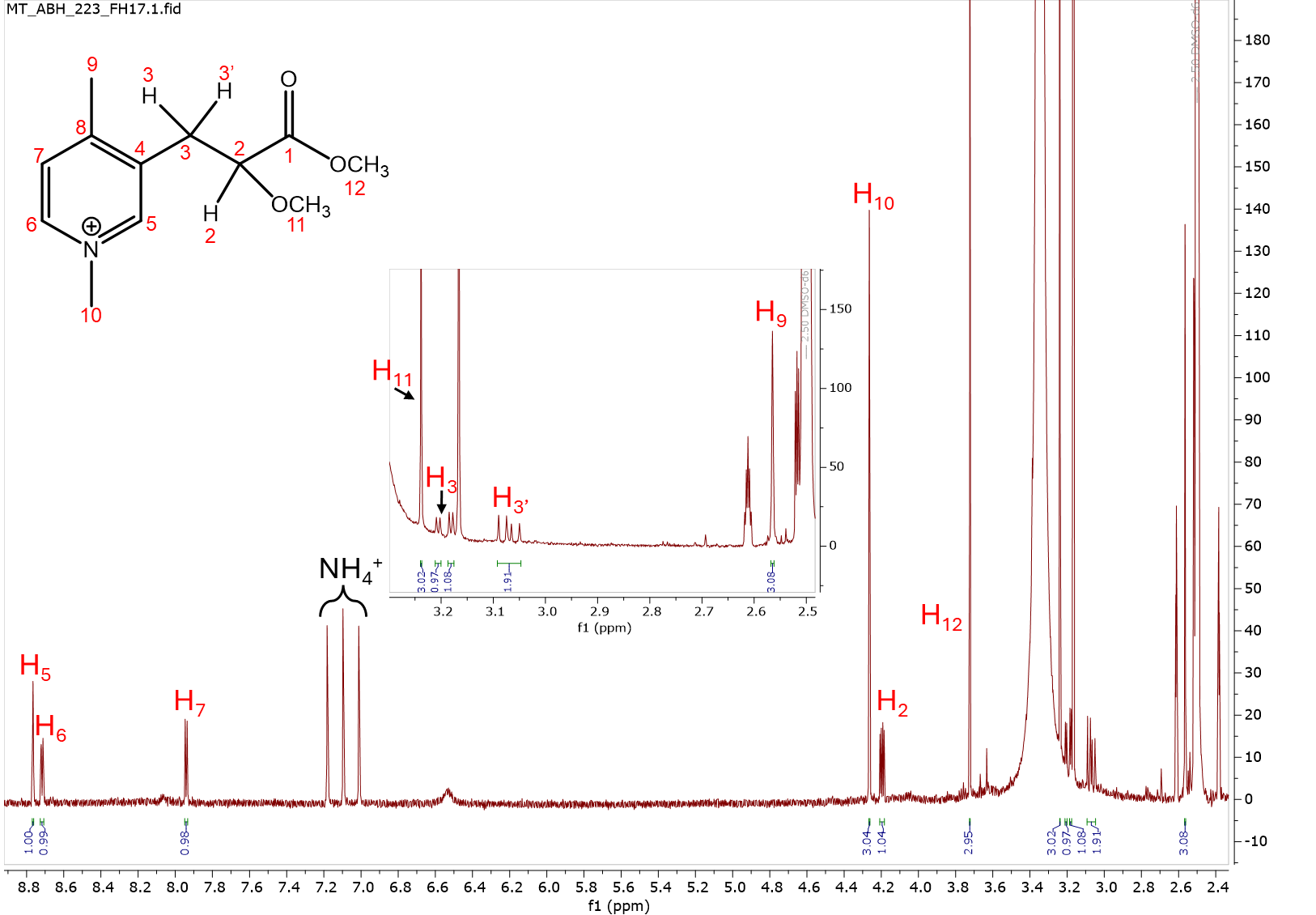


**Figure S17:** ^1^H-NMR spectrum of **4** in DMSO-*d*_6_ at 600 MHz


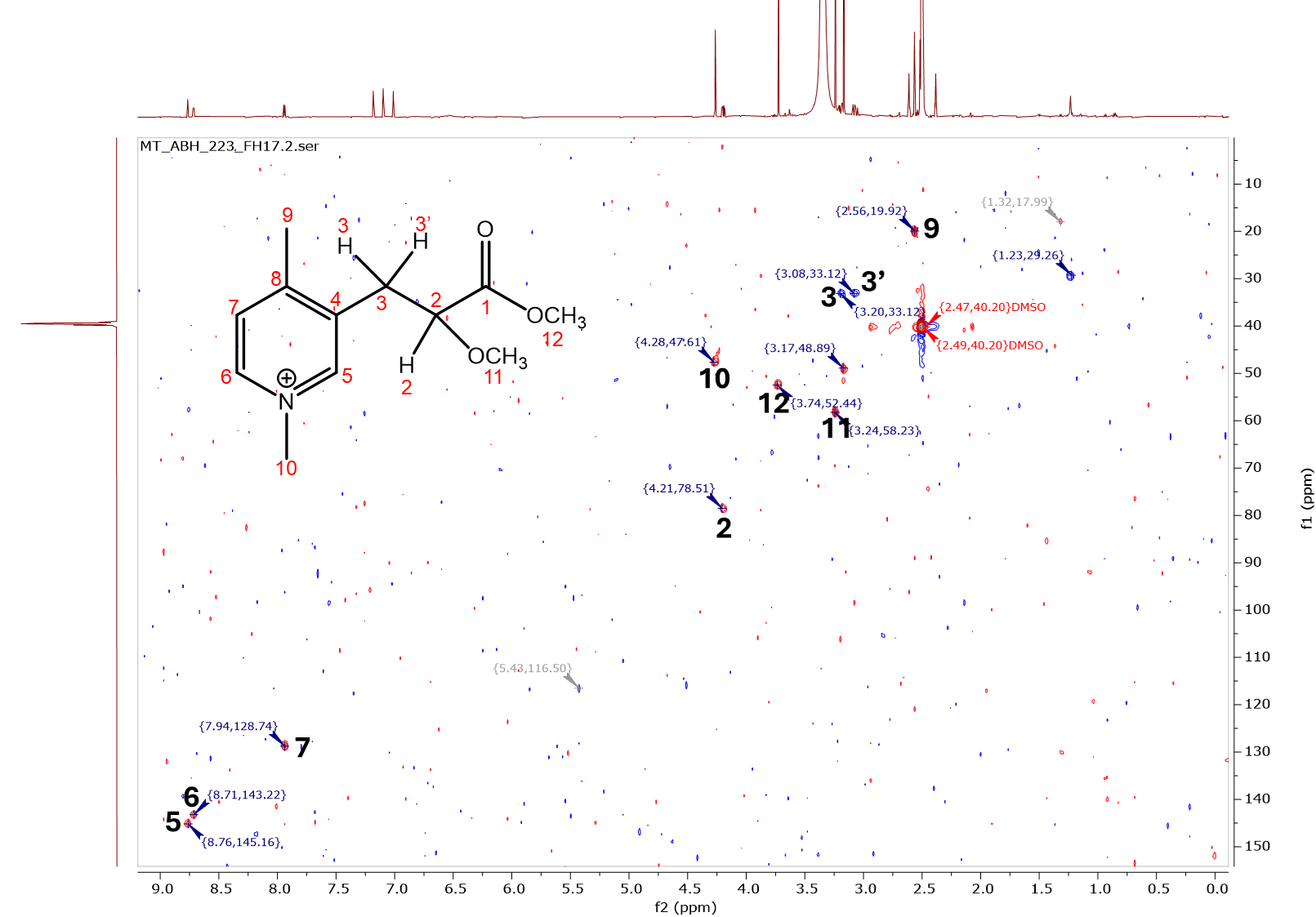


**Figure S18:** HSQC spectrum of **4** in DMSO-*d*_6_ at 600 MHz.


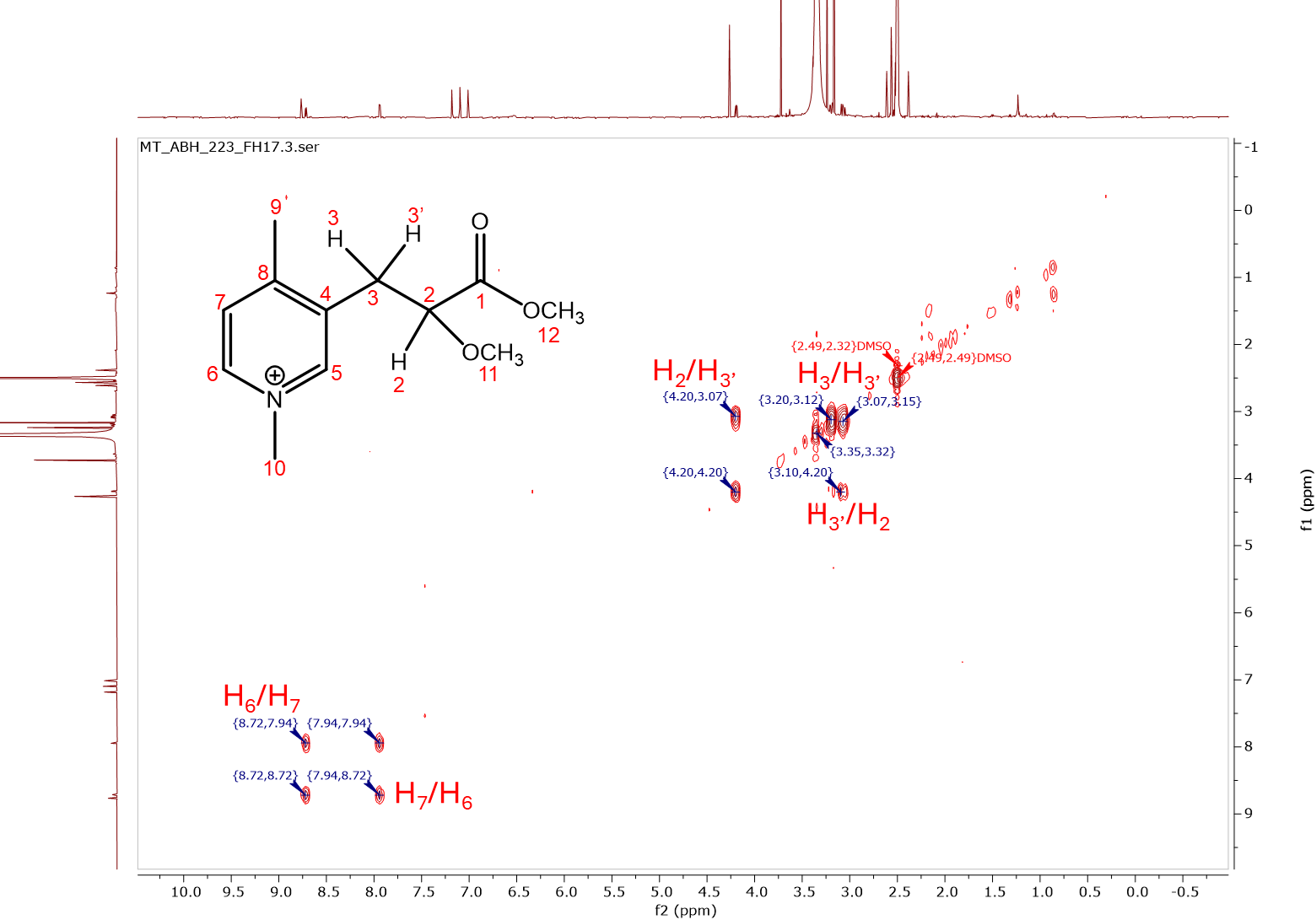


**Figure S19:** COSY spectrum of **4** in DMSO-*d*_6_ at 600 MHz.


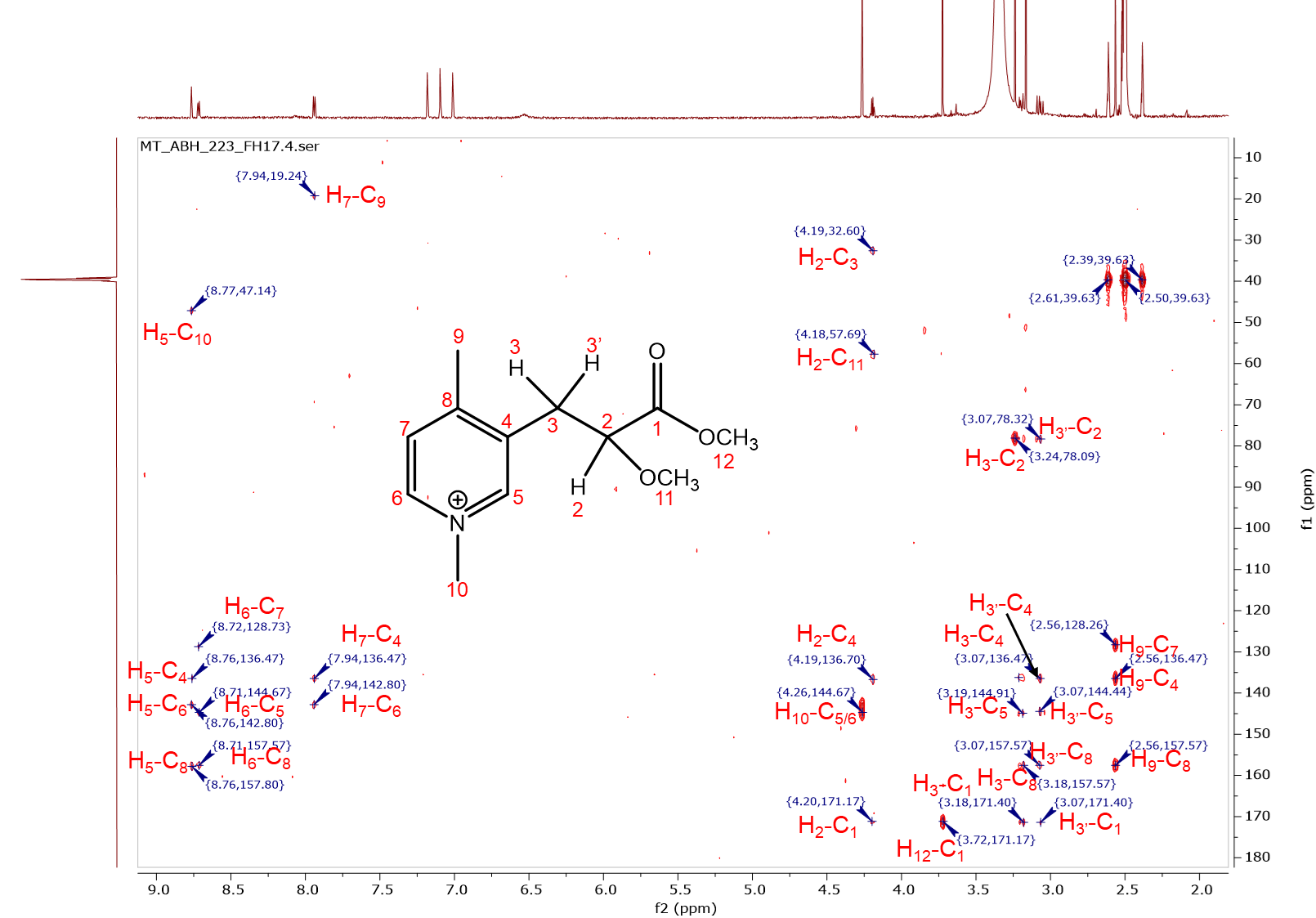


**Figure S20:** HMBC spectrum of **4** in DMSO-*d*_6_ at 600 MHz.

**Table S2:** 600 MHz NMR data for sulcatin A-C (**2-4**) in DMSO-*d*_6_.

| **Position** | **2** | | | | **3** | | **4** | |
| --- | --- | --- | --- | --- | --- | --- | --- | --- |
|  | **^13^C δ^*^** | **^1^H δ (mult, *J* Hz)** | **COSY** | **HMBC**  **(^1^H → ^13^C)** | **COSY** | **HMBC**  **(^1^H → ^13^C)** | **COSY** | **HMBC**  **(^1^H → ^13^C)** |
| 1 | 173.3 |  |  |  |  |  |  |  |
| 2 | 32.0 | 2.66 (t, 7.8, 2H) | 3 | 1,3,4 | 3,3' | 1,3,4,11 | 3' | 1,3,4,11 |
| 3 | 24.5 | 2.97 (t, 7.8, 2H) | 2 | 1,2,4,5,8 | 3',2 | 1,2,4,5,8 | 3' | 1,2,4,5,8 |
| 3’ |  |  |  |  | 3,2 | 1,2,4,5,8 | 2,3 | 1,2,4,5,8 |
| 4 | 139.6 |  |  |  |  |  |  |  |
| 5 | 143.6 | 8.78 (s) | 6 | 3,4,6,8,10 | 10 | 3,4,6,8,10 |  | 8,6,4,10 |
| 6 | 142.0 | 8.68 (d, 6.3) | 5,7 | 5,8 | 7 | 5,7,8,10 | 7 | 5,7,8 |
| 7 | 127.8 | 7.91 (d, 6.3) | 6 | 9,4,6 | 6,9 | 4,6,9 | 6 | 5,6,9 |
| 8 | 156.9 |  |  |  |  |  |  |  |
| 9 | 19.0 | 2.57 (s, 3H) |  | 4,7,8 | 7,10 | 4,7 |  | 4,7,8 |
| 10 | 46.6 | 4.24 (s, 3H) | 9 | 5 | 5,9 | 5,6 |  | 5,6,8 |
| 11 |  |  |  |  |  | 2 |  | 2 |
| 12 |  |  |  |  |  |  |  | 1 |

*obtained from HSQC and HMBC spectra, respectively


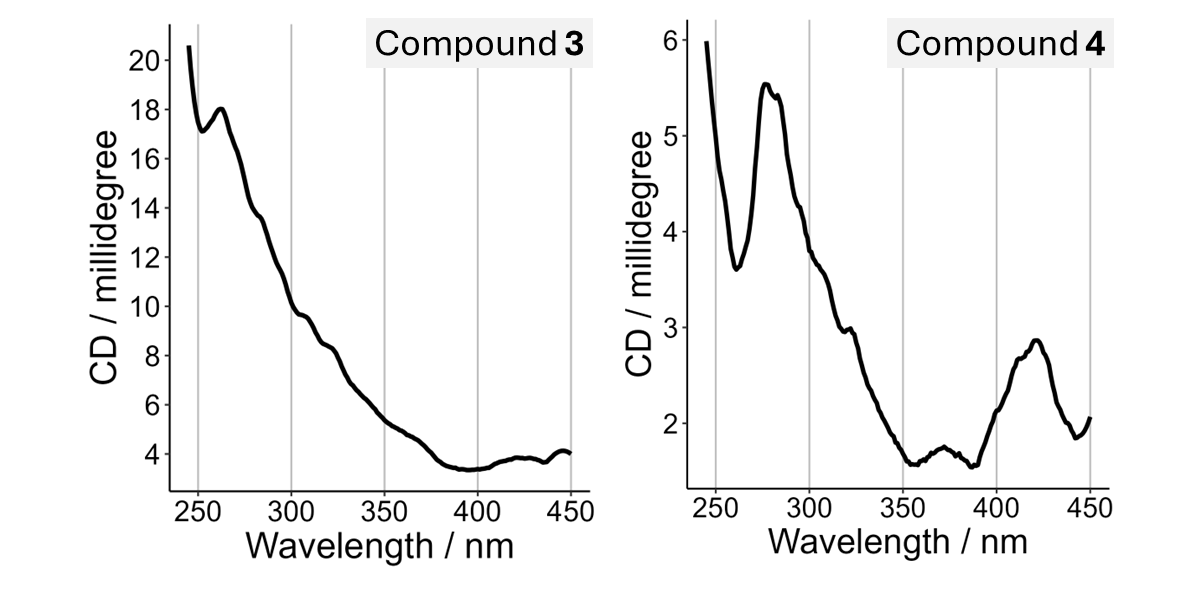


**Figure S21:** Circular dichroism spectra for sulcatin B (**3**) and sulcatin C (**4**), both displaying positive Cotton effects

**Figure S22:** First cell-free, tau-tau binding experiment. DMMTC run as a positive control and its activity was within the normal range, B_50_ = 6.92 µM (A); asterubine (**1**), sulcatin A (**2**), sulcatin B (**3**), and sulcatin C (**4**) (B-E) were found to be inactive up to 500 µM. Datapoints represent the mean ± standard error (n = 4).


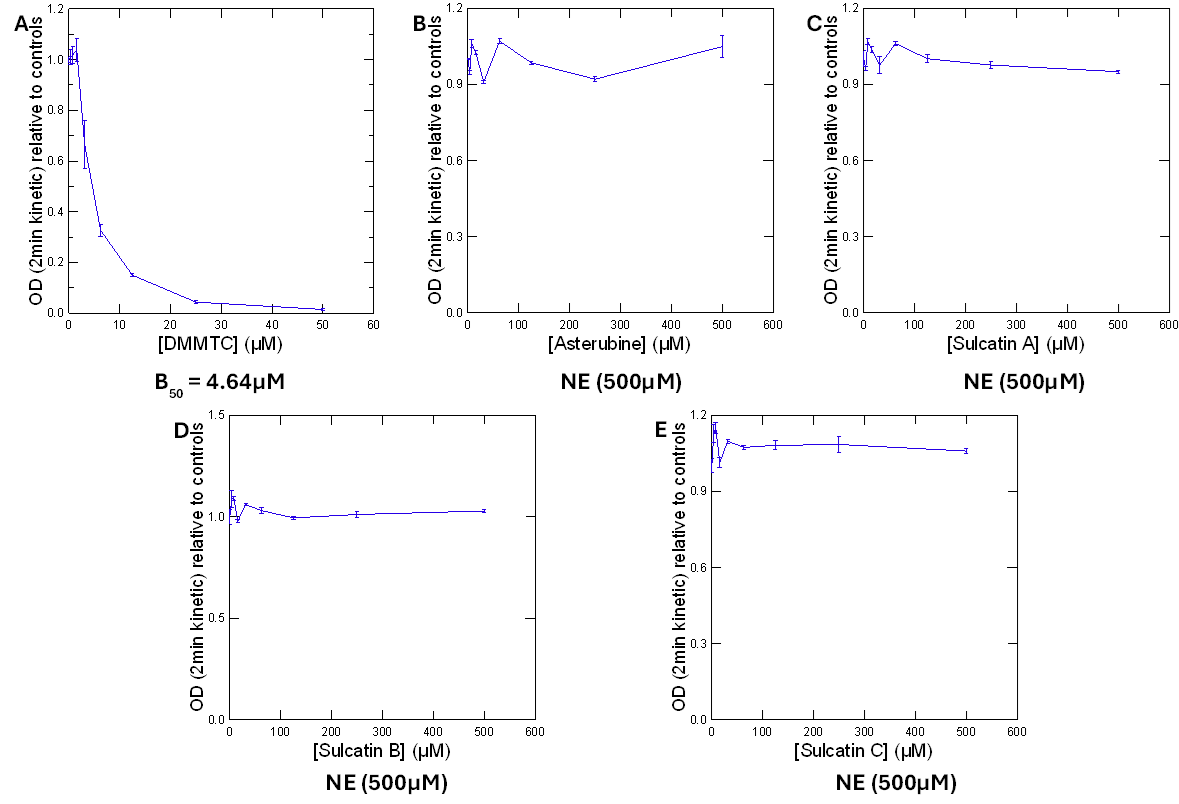


**Figure S23:** Second cell free tau-tau binding experiment. DMMTC included as a positive control and its activity was within the normal range, B_50_ = 4.64 µM (A); asterubine (**1**), sulcatin A (**2**), sulcatin B (**3**), and sulcatin C (**4**) (B-E) were found to be inactive up to 500 µM. Datapoints represent the mean ± standard error (n = 4).


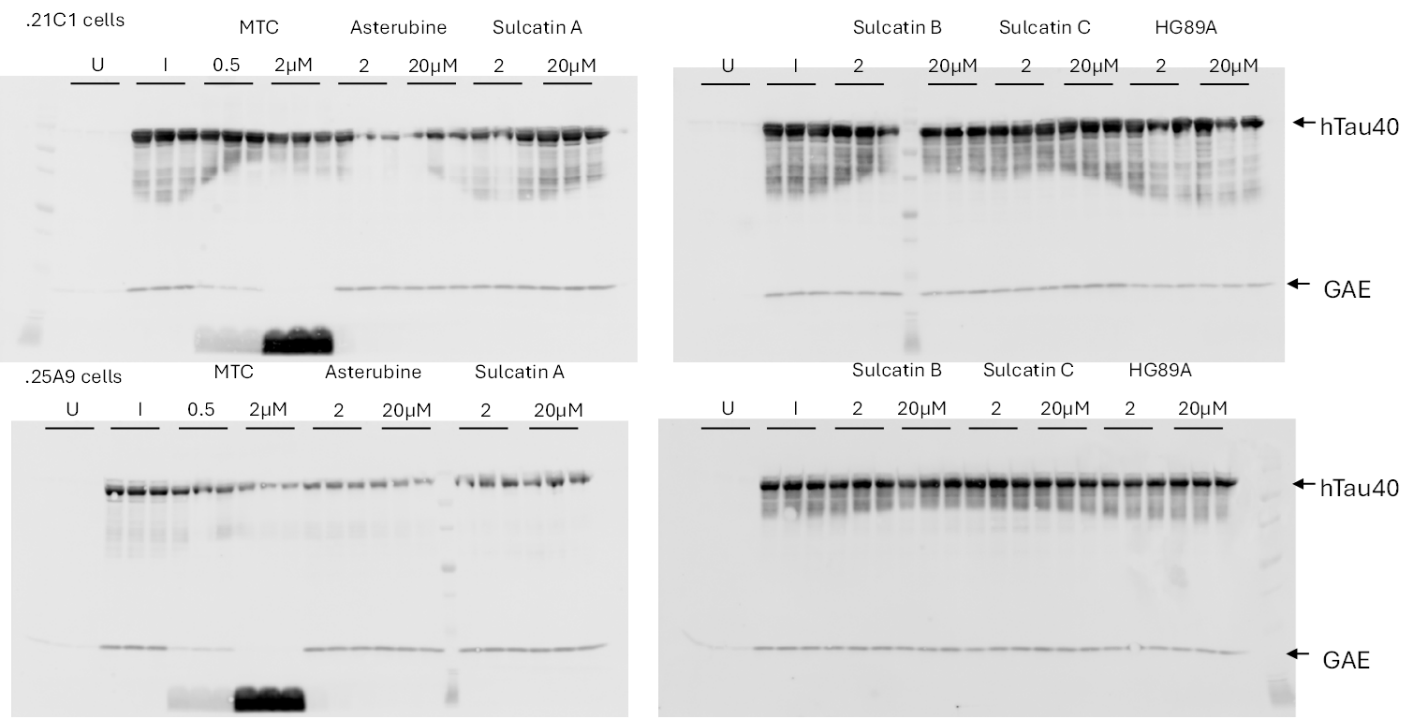


**Figure S24:** Western blots of samples tested using clone 21C1 (top) and 25A9 (bottom). Tau protein labelled with mAb 7/51. MTC was included as a positive control. U = no induction of hTau40. All other cells were induced with 100µM IPTG. I = induced but not treated with any compound.


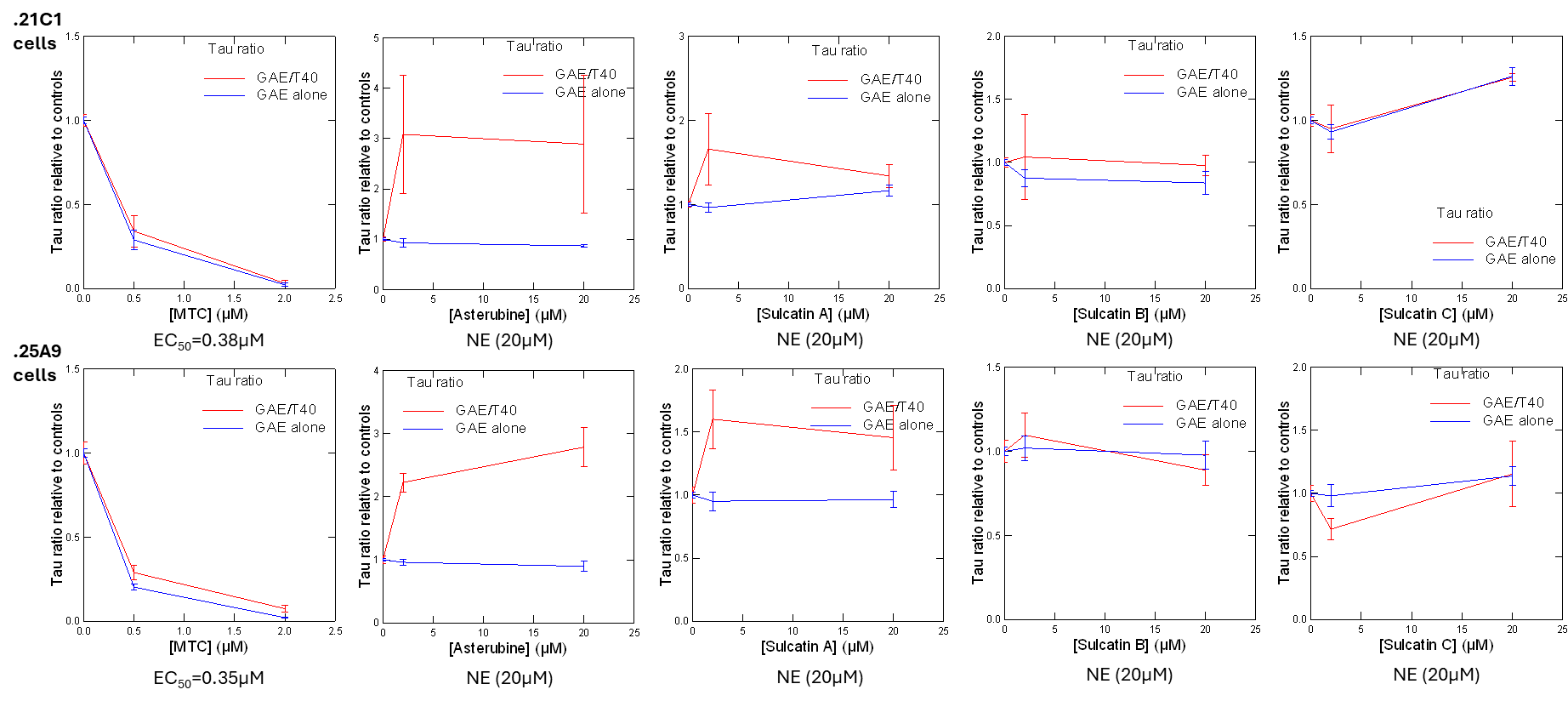


**Figure S25**: Effect of compounds in cell-based tau aggregation assay. The ratios of tau bands (either GAE/hTau40 [red] or GAE alone [blue]) relative to induced cells but not treated with compound. The EC_50_ value is the concentration of compound at which the GAE/hTau40 ratio = 0.5. MTC was active in both cell clones with EC50 values of 0.38 and 0.35µM for clone 21C1 (upper panel) and 25A9 (lower panel), respectively. Asterubine (**1**), sulcatin A (**2**), sulcatin B (**3**), and sulcatin C (**4**) were inactive at the maximum concentration tested of 20µM. Datapoints represent the mean ± standard error (n = 3).
